# Standards of care for pediatric clinical service delivery: A rapid scoping review

**DOI:** 10.1177/13674935251355056

**Published:** 2025-07-03

**Authors:** Brittany Barber, Annette Elliott-Rose, Amanda Higgins, Kristy Hancock, Amy Mireault, Katie McDonald, Stacy Burgess, LeeAnn Larocque, Christine Cassidy

**Affiliations:** 1 School of Nursing, 3688 Dalhousie University, Halifax, NS, Canada; 2 IWK Health, Halifax, NS, Canada; 3 Maritime SPOR SUPPORT Unit (MSSU), Nova Scotia Health, Halifax, NS, Canada

**Keywords:** standards of care, pediatric, levels of service, rapid scoping review, care coordination

## Abstract

When pediatric services are not coordinated, children, youth, and their caregivers cannot always access the right services at the right time. Guidance is needed for how pediatric services should be planned and organized to improve integrated care. The objective of this rapid scoping review was to map and characterize current evidence on standards of care for integrated pediatric levels of service. This rapid scoping review was conducted in accordance with Cochrane Rapid Reviews Interim Guidance. We searched academic databases and gray literature in 2022, published in English from Canada, United States, United Kingdom, Australia, New Zealand, with no date parameter. Fifty-three sources met inclusion criteria and were included in this review. Levels of service frameworks categorized services into three, four, or six distinct levels. Eight sources described integrated levels of service. Most pediatric standards of care frameworks defined levels of service by roles and responsibilities. Differences were defined by transitions between levels of care, planning for services across urban and rural communities, and coordinating integrated levels of service. Future research is required to build evidence base of how levels of service frameworks can be used in practice, adapted to local contexts, and evaluated.

## Introduction

Access to comprehensive and coordinated care is critical for delivering quality pediatric care (American Academy of Pediatrics et al., 2012). Children and youth (younger than 18 years of age) and their caregivers are often challenged with managing and coordinating care within health systems that silo pediatric clinical services by subspecialty and function independently of one another with varying levels of care (Meehan et al., 2019). When pediatric health services are not integrated across care settings, children and their caregivers cannot access the right services at the right time and experience worse health outcomes (Allen et al., 2009). A lack of integrated care also presents challenges for decision-makers to allocate resources efficiently and effectively with equitable standards of care that meet health needs of pediatric patients across urban and rural settings (Canadian Institutes of Health Research, 2020; Leatt et al., 2000; Wolfe et al., 2020).

When health services are integrated through co-location of services and sharing of information, they have the potential to generate significant health benefits, including improved quality patient-centered services, reduced health system costs, and meeting continuum of care needs (Armitage et al., 2009; Kelly et al., 2020; Shortell et al., 1994; Suter et al., 2009; World Health Organization, 2016). Integrated care has been defined through functional integration (coordination of financial and information management and human resources), care provider integration (collaborative relationships between care providers and extent that care providers are economically linked to a system and actively participate in its planning and management within leadership positions), and clinical integration (continuity and coordination of care, disease management, and sharing of information) (Conrad and Shortell, 1996; Devers et al., 1994; Leatt et al., 2000). Health systems commonly achieve integrated care through horizontal and vertical integration of services (World Health Organization, 2016). Horizontal integration occurs when health organizations delivering the same level of service come together (Heeringa et al., 2020; World Health Organization, 2016). For example, a single specialty group practice would merge into a team of multidisciplinary healthcare professionals delivering similar services at one collaborative care clinic (Mezzalira et al., 2024). Vertical integration occurs when health organizations or programs delivering different levels of care come together (e.g., primary care, acute care, post-acute care) (Heeringa et al., 2020; World Health Organization, 2016). For example, different levels of care merge within one acute care hospital setting to provide a range of general and subspecialty care including pharmacy services, diagnostic imaging and laboratory services, and post-acute rehabilitation (Amado et al., 2022).

When pediatric health services are integrated, children and their caregivers have improved access to healthcare services and quality of care as close to home as possible. Pediatric-specific integrated models of care improve quality of life, reduce health care use and system costs, lower family expenses by minimizing travel, enhance communication between providers and caregivers, and strengthen family-centered plans that address medical and social needs of children and caregivers (Adams et al., 2013; Allen et al., 2009; Satherley et al., 2021; Wolfe et al., 2020). For example, when pediatric mental health care services are integrated within collaborative care teams, children and their caregivers have increased access to behavioral health treatment and experience improved mental health outcomes (Burkhart et al., 2020). Achieving integrated pediatric care remains challenging due to fragmented funding models, disconnected administrative, organizational, and service structures (Cumming, 2011), and the complexity of coordinating services for children with diverse health conditions across different developmental stages (Cohen and Coller, 2020; O’Shea et al., 2018). Improving integrated care is particularly important for children and youth with medical complexity that require subspecialty care and more frequent use of primary health care services, hospital care, and emergency department care for chronic conditions such as malignant neoplasms, cardiovascular diseases, and gastrointestinal diseases (Adams et al., 2013; Canadian Institutes of Health Research, 2020).

Coordinating integrated care from multidisciplinary teams is challenging for children at different developmental stages and across different community settings. Multidisciplinary care teams commonly experience time constraints for interprofessional collaboration, inadequate training in collaborative care settings, frequent turnover of professionals, and meeting specialized needs of children while addressing family-centered needs of multiple caregivers (Nooteboom et al., 2021). Further, planning and coordinating integrated pediatric care across different community settings is challenging for communities with different levels of service available to meet pediatric population health needs. Countries with large geographical territories spanning urban and rural communities must coordinate levels of service across rural or remote communities with low population density and fewer resources to deliver more specialized care (Waibel et al., 2021).

Health systems have developed frameworks, or standards of care for service delivery, to guide how health care services should be organized and coordinated at medical centers including responsibilities and requirements needed to deliver high-quality care based on population need and health care resources (Waibel et al., 2021). For example, hospital health care services are routinely defined within three levels of service (Table 1) (McCord et al., 2015; Rosenberg and Moss, 2004). It is unclear how standards of care frameworks define and operationalize integrated levels of service specific to pediatric populations.

**Table 1.** Levels of service definition and terminology.

Levels of service	Definition and terminology	
Level 1	Hospital with 50–250 beds and few specialties, covering general practice areas of internal medicine, obstetrics and gynecology, pediatrics, and general surgery. Often only one general practice physician or nonphysician clinician and limited laboratory services available for general analysis but not for specialized pathological analysis.	Commonly referred to as: *-Primary-level hospital* *-District hospital* *-Rural hospital* *-Community hospital*
Level 2	Hospital with 200–800 beds and differentiated by function to deliver more specialized care with as many as 5 to 10 clinical specialties.	Commonly referred to as: *-Regional hospital* *-General hospital*
Level 3	Hospital with 300–1500 beds and highly specialized staff and technical equipment (e.g., cardiology, intensive care unit, specialized imaging units). Clinical services are highly differentiated by function and serve as an academic or university hospital by providing teaching activities.	Commonly referred to as: *-National hospital* *-Central hospital* *-Academic, teaching, or university hospital*

Source: (McCord et al., 2015; Rosenberg and Moss, 2004).

An overview of evidence defining standards of care and integrated levels of service is critical for decision-makers. Integrated levels of service frameworks are needed to provide a guide for systems planning across organizations and geographical boundaries. For example, it could allow decision-makers to promote active health service planning such as allocating appropriate resources, staffing, roles, equipment, supports, etc., needed to provide timely and equitable access to care based on available financial and health human resources at local, regional, and provincial levels. Integrated and coordinated levels of service also promote quality and patient safety while reducing redundancies and inconsistencies in care. For example, integrated care could enhance delivery of person and family-centered services by matching the appropriate level of care with pediatric needs and coordinating commonly accessed services like primary care as close to home as possible.

## Aim

To map and characterize current evidence on standards of care for integrated pediatric levels of service. Our objectives were to:

1. Identify standards of care for pediatric clinical service delivery
2. Describe how pediatric levels of service are defined and integrated

## Methods

This rapid scoping review was conducted in accordance with Cochrane Rapid Reviews Interim Guidance from Cochrane Rapid Reviews Methods Group (Garritty et al., 2020).

## Inclusion criteria

The most recent literature and jurisdictional review (Waibel et al., 2021) was conducted to develop a framework for defining and planning pediatric services across British Columbia, Canada. This review focused on a limited search of academic peer-reviewed literature published within MEDLINE since 2008. Our rapid scoping review expands a search to include multiple academic and gray literature sources of pediatric clinical services without a date parameter.

This rapid scoping review considered quantitative, qualitative, and mixed method study design for inclusion. Systematic reviews of text and opinion were also considered for inclusion in the proposed scoping review. Articles published in English language were included, based on language capabilities of our review team. There were no date parameters on sources that met inclusion criteria for this review to ensure a comprehensive understanding of both historical foundations and contemporary practices, capturing the full scope of standards of care. This review included academic and gray sources of literature identifying pediatric standards of care for coordinating and delivering clinical services across different levels of service. The search strategy targeting both academic and gray literature sources aimed to retrieve all sources related to standards of care and levels service delivery available on a variety of platforms including peer-reviewed journals and healthcare organization websites. A description of operational terms and definitions is summarized within Table 2.

**Table 2.** Operational terms and definitions.

Terms	Definitions
Pediatric patients	In **Canada**, a child is referred to as a pediatric patient if they are under the age of 16, and a youth if they are older than 16 years of age but under age of majority, which is 18 or 19 years of age depending on provincial jurisdictions (Canadian Paediatric Society, 2018). In **Australia**, pediatric patients are considered if they are under age of 13, and a young person if they are older than 13 but under age of 22 (Clark et al., 2015). In **New Zealand**, pediatric patients are considered under age of 16 years, and adolescent or young adult patients aged 10 to 24 years (Starship Child Health, 2021). In **United Kingdom**, pediatric patients are considered under age of 18 years, and young adults if they are from age of 16 to 18 years (National Health Service, 2020).
Integration of care	A health system understanding of integration of care is defined as health services that are managed and delivered across different levels and sites of care within health systems and transitioning into community settings. The purpose of integration of care is to coordinate the right care at the right time according to an individual’s needs across the life course, so that care exists across a continuum of health promotion, disease prevention, diagnosis, treatment, disease management, rehabilitation, and palliative care services (Contandriopoulos et al., 2003; Goodwin, 2016).
Standards of care	A standard of care refers to informal and formal guidelines, measures or quality statements established nationally and generally accepted by healthcare providers for treatment of a disease or condition (Australian Commission on Safety and Quality in Health Care, 2014). Standards of care can be clinical practice guidelines, a formal diagnostic or treatment process, or a protocol for delivering care based on evidence of best practice (Chew et al., 2016).

### Population

This review is focused on pediatric clinical services designed and delivered for children and youth younger than 18 years, age of majority for many countries including Canada, Australia, and United Kingdom (UK).

### Concept

This review includes literature exploring pediatric standards of care and levels of service coordinated across regions with varying resources. Coordination of care is particularly a challenge for countries with large geographical territories spanning urban and rural communities, as rural or remote communities with low population density have fewer resources delivering level or tier one services in comparison to densely populated cities with greater resources to deliver higher and more complex levels of service.

### Context

This review includes literature focusing on delivery of pediatric services across different clinical areas including oncology, palliative care, general, emergency, maternal and newborn health, and other subspecialty care areas. During data extraction and data presentation phases, we documented what pediatric clinical service areas are included in the evidence.

### Search strategy

Our search strategy aimed to locate published peer-reviewed literature and gray literature. A list of academic and gray literature sources, keywords, and index terms were developed by all team members in conjunction with a health sciences librarian. A search of databases was executed by a health sciences librarian on our research team.

First, a limited search of MEDLINE (Ovid) was conducted to identify academic sources on this topic. Text words contained in titles and abstracts of relevant articles (Ogle et al., 2018; Rosenberg and Moss, 2004; Waibel et al., 2021) and index terms describing articles were used to develop a full search strategy for MEDLINE including variations of (*child* OR *adolesc* OR *pediat* OR *paediat)*; (*standard?* OR *level?* OR *tier* OR *framework)*; (*care* OR *service?* OR *health)*; (standard of care); (*integrat* OR *coordinat* OR *comprehensive*); (*care* OR *service?* OR *health*); (*delivery of health care)* (see Supplemental Table I).

Our search was developed using three main concepts: pediatrics, standards of care, levels of care, and integration or coordination of care. A pediatric search filter (Tjosvold et al., 2020) was modified for our needs and used to define pediatrics. A MEDLINE search was translated to Embase (Elsevier), Cumulative Index of Nursing and Allied Health (CINAHL) Full Text (EBSCO), and Scopus (Elsevier) and run on June 30, 2022 from inception (see Supplemental Table I for all database searches). Due to time constraints and in line with Cochrane’s rapid review guidance, our database searches were not peer-reviewed by a second health sciences librarian. Reference lists of included academic articles included were manually screened and a forward search of article titles that met inclusion criteria was conducted by one reviewer.

Google was used to source gray literature from Canada, UK, New Zealand, Australia, and United States (US). Focusing on selected high-income countries allows for an in-depth search of sources and comparison between countries with similar tiered health systems and resources for delivering services across large geographical areas (Canadian Institutes for Health Information, 2019). Two different Google searches (Supplemental Table II) were run five times (10 searches total), using country search filter on Google’s advanced search page. Sources from first five pages of results were screened, and relevant websites uploaded to an Excel document. Gray literature sources included websites, organizational reports, policy literature, and government documents.

### Study selection

**Figure 1.**
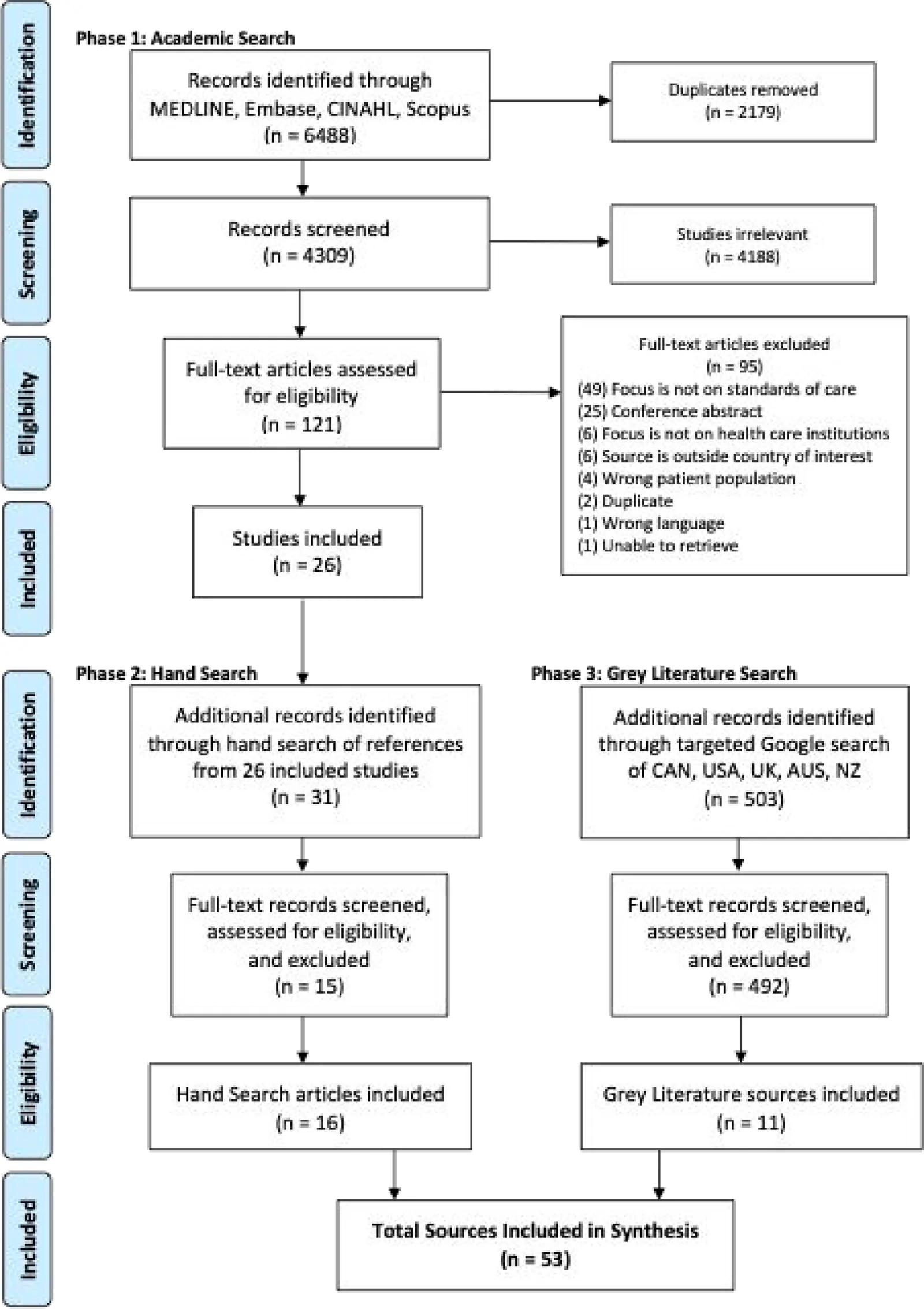
Prisma flow diagram.

*Phase 1:* All records identified through database searches were uploaded into Covidence (Covidence Veritas Health Innovation Ltd, 2023), a screening management system, and duplicate records removed. Title and abstracts were screened independently by two reviewers for assessment against the eligibility criteria. Full texts of potentially relevant articles were uploaded into Covidence and assessed in detail against inclusion criteria by one reviewer (Garritty et al., 2020). Results are reported using the Preferred Reporting Items for Systematic Reviews and Meta-analyses (PRISMA) checklist (Tricco et al., 2018) and presented within Figure 1 PRISMA flow diagram (Page et al., 2021). Reason for exclusion of full text articles that did not meet inclusion criteria are reported in Figure 1.
*Phase 2:* Reference lists of included academic sources were hand searched by one reviewer to identify additional sources with titles that included key terms such as pediatrics, standards or guidelines of care, levels of service, and integration or coordination of care. The citation information for potentially relevant sources was recorded within an Excel document for full text review. Full text articles were assessed against eligibility criteria by one reviewer.
*Phase 3:* Google searches were split between two different reviewers. Each reviewer ran their searches and screened first five pages of results directly in Google. Citation information of potentially relevant sources was added to an Excel document and duplicates manually removed. Full text sources were assessed against inclusion and exclusion criteria by one reviewer. All sources with sufficient information on pediatric standards of care and levels of service were included. Gray literature sources included websites, organizational reports, policy literature, and government documents. Sources that did not focus on pediatric standards of care or did not provide enough information for data extraction were excluded.

### Data extraction

Data were extracted from each source by two independent reviewers using our data extraction tool developed by the research team (Supplemental Table III: Data Extraction Instrument). Extracted data included specific details about author(s); year of publication; title of source; country of origin; study aim/purpose; study population (children age, diagnoses); pediatric clinical service area; study setting (healthcare/ clinical setting); study design; guidelines of care; reported health systems outcomes; integration with other services; and implementation strategy (barriers or facilitators).

### Analysis

A narrative synthesis of data extraction results was conducted, integrating key characteristics of included sources to develop a comprehensive overview of the evidence base. Findings were categorized by pediatric clinical service areas and healthcare settings, enabling identification of trends in care guidelines, levels or tiers of service, integration approaches with other services, and associated health system outcomes.

## Results

### Pediatric standards of care

Fifty-three sources met inclusion criteria for this review, including 42 academic articles and 11 gray literature sources (Figure 1 PRISMA). Pediatric standards of care were developed by US (n = 18), UK (*n* = 13), Canada (*n*= 11), Australia (*n* = 7), New Zealand (*n* = 3), and International Federation of Emergency Medicine (*n* = 1). Identified sources reported on pediatric clinical service area general pediatrics (*n* = 7), maternal and newborn health (*n* = 7), oncology (*n* = 6), palliative care (*n* = 6), emergency (*n* = 4), pediatric intensive care unit (PICU) (*n* = 4), mental health (*n* = 3), dentistry (*n* = 3), and subspecialty care (*n* = 13) (e.g., diabetes, language difficulties and disorders, bladder and bowel). A summary of findings for 27 sources reporting levels of service are presented in Table 3 and a complete summary of findings for 53 sources are presented in Supplemental Table IV.

**Table 3.** Academic and gray literature results describing pediatric levels of service.

Author	Title	City	Clinical service area	Study aim/purpose	Description of standard or guideline	Description of levels or tiers of service
(Barnea et al., 2021)	From fragmented levels of care to integrated health care: Framework toward improved maternal and newborn health.	USA	Maternal and newborn health	Develop an integrated tiered model of care that leverages a multidisciplinary team to deliver a coordinated set of perinatal healthcare services based on the mother’s associated co-morbidities and level of risk.	Recommended standards of care for integrated maternal care includes timely access to care and counseling, coordinated care for pregnancy and postpartum, protocols for on-going assessment and transfer to high level care, and hospital level of care based on trained personnel. Resources for maternal care at point of entry into care enables risk assessment (e.g., low, moderate, and high risk) and a plan for integrated care to start right away. Timely risk assessment minimizes maternal-newborn dyad outcome.	**4 levels of obstetric care**: Level I: Low-risk uncomplicated pregnancy, well-controlled gestational diabetes, mild pre-eclampsia, twins Level II: Level I plus additional risk factors like complex cesarean delivery, medical complications requiring close monitoring (i.e., asthma, chronic hypertension, diabetes) Level III: Level II plus additional factors like maternal complications (cardiac, coagulation disorders), immune disorders, placental pathologies (accrete, previa, bleeding), severe preterm pre-eclampsia Level IV: Level III plus additional factors like severe maternal disease (cardiac, pulmonary), maternal disease requiring complex surgery, unstable maternal condition
(Darling et al., 2014)	Implementation of the Canadian Paediatric Society’s hyperbilirubinemia guidelines: A survey of Ontario hospitals	CAN	Maternal and newborn health	Examine whether hospitals implemented neonatal standard of care guidelines and investigate how guideline recommended care is organized, including factors influencing guideline implementation.	Recommended standard of care includes universal neonatal bilirubin screening, timely follow-up for babies requiring repeat bilirubin testing after hospital discharge, and follow-up from community-based care providers including pediatricians, family physicians, midwives, and nurse practitioners.	**3 levels of neonatal care**: Level 1: Basic level of service Level 2: Intermediate care Level 3: Specialized level of service
(Ebbels et al., 2017)	Evidence-based pathways to intervention for children with language disorders	UK	Subspecialty (language difficulties and disorders)	Examine evidence regarding effectiveness of interventions for children with language disorders at different tiers and to propose an evidence-based model of speech and language therapy service delivery to aid clinical decision-making.	A tiered approach to service delivery is currently recommended, whereby services become increasingly specialized and individualized for children with greater needs. Pediatric speech and language therapist roles often involve planning individualized interventions for specific children and contribute to delivery of care at each tier 1, 2, and 3 of services.	**3 tiers of service:** Tier 1: Universal services with high-quality teaching for all Tier 2: Targeted services providing education-led language programs Tier 3: Specialist services providing individualized interventions delivered by speech and language therapists
(Finella et al., 2007)	IMPaCCT. Standard pediatric palliative care in Europe	UK	Palliative care	Compare pediatric palliative care, best practices, and minimum standards agreed. IMPaCCT recommends these standards become implemented in all European countries.	IMPaCCT recommends following minimum core standards of care for Europe. 1. Provision of care, 2. Unit of care, 3. Care team, 4. Care coordinator/Key worker, 5. Symptom management, 6. Respite care, 7. Bereavement, 8. Age-appropriate care, 9. Education and training 10. Funding for palliative services 11. Euthanasia, 12. Symptom recognition and assessment, 13. Core principles of symptom management, 14. Core principles of pain management	**3 levels of pediatric palliative care:** Level 1: A palliative care approach. Palliative care principles should be appropriately applied by all healthcare professionals Level 2: General palliative care. At an intermediate level, a proportion of patients and families will benefit from expertise of healthcare professionals who, although not engaged full time in palliative care, have had some additional training and experience in palliative care. Level 3: Specialist palliative care. Specialist palliative care services are those whose core activity is provision of palliative care.
(Hsu et al., 2019)	Executive summary: Criteria for critical care of infants and children: PICU Admission, discharge, and triage practice statement and levels of care guidance.	USA	PICU	Executive summary of guidelines and levels of care for PICU to address transformation of the field and summarize a revised set of guidelines that will enable hospitals, institutions, and individuals in developing appropriate PICU for their community needs.	An update of American Academy of Pediatrics and Society of Critical Care Medicine’s 2004 guidelines and levels of care for PICUs. Specific recommendations include 1. PICU level of care admission criteria, 2. ICU structure and provider staffing model, 3. ICU personnel and resources, 4. Performance improvement and patient safety, 5. Equipment and technology, 6. PICU discharge and transfer criteria.	**3 levels of PICU care:** Level 1: Community-based PICU centers providing level II PICU care with a broad range of services and resources and located in general medical-surgical institutions with capability of treating pediatric patients Level 2: Tertiary PICU centers providing level II advanced care for many medical and surgical illnesses in infants and children Level 3: Quaternary or specialized PICU facilities providing regional comprehensive care and serving large populations with complex patients. Highest level PICU facility supporting level I and level II centers.
(Ogle et al., 2018)	Levels of type 1 diabetes care in children and adolescents for countries at varying resource levels.	AUS	Subspecialty (diabetes)	Develop a “levels of care” framework for young people with type 1 diabetes to help facilitate the work of policy makers, health planners, professional and lay advocates, and care providers in countries where comprehensive guideline-based care is mostly inaccessible.	Guidelines of care include services delivered within tiers including minimal, standard, and comprehensive care. Within each level details on insulin therapy, blood glucose monitoring, HbA1c testing, complications screening and diabetes education and multidisciplinary team care are provided.	**3 tiers of service:** Tier 1: Lowest level, designated 1A, human insulin (if available), is given through two injections daily. Tier 2: Intermediate care is multiple daily injections (“basal bolus regimen”), accompanied by HbA1c testing and diabetes education provided by a pediatric or adult endocrinologist and/or a diabetes nurse educator. Tier 3: Comprehensive, guidelines-based care.
(Rogers, 2020)	Developing a community-based integrated children’s bladder and bowel service.	UK	Subspecialty (bladder and bowel)	Develop an integrated children’s bladder and bowel service to ensure affected children are seen at the right time and place and by the right person.	Guidelines for delivering children’s bladder and bowel care should focus on appropriate level of service (level 1 and 2) and integrated services between general health services and community-based care to improve self-management of care.	**2 levels of care:** Level 1: Universal services provided by health visitors, nursery nurses and school nurses, who all potentially play an important role in early identification and first-line management of bladder and bowel problems, such as delayed toilet training, bedwetting, and onset of constipation. Level 2: Nurse-led, community-based, children’s bladder and bowel (continence) service. This service would undertake comprehensive bladder and bowel assessments and treat children and young people from 0–19 years with bladder and/or bowel problems, and/or delayed toilet training, where universal level 1 interventions have not resolved the problem
(Rothschild and Derrington, 2020)	Palliative care for pediatric intensive care patients and families.	USA	Palliative care	Review of evidence and best practices for integrating palliative care into pediatric intensive care unit (PICU) with a focus on surgical patients.	Palliative care is best integrated in a tiered approach with primary palliative care provided by PICU and surgical providers including basic symptom management, high-quality communication, and end-of-life care. Secondary and tertiary levels of care involve unit or team-based champions with subspecialty care teams.	**3 levels of service:** Level 1: Primary palliative care provided by members of primary teams, considered part of core competencies for the field. Level 2: Secondary palliative care provided by “champions” within primary team who have additional expertise and/or training in palliative care principles, champions collaborate often with subspecialty palliative care team Level 3: Tertiary palliative care provided by the subspecialty palliative care team
(Waibel et al., 2021)	Development of the tiers of service framework to support system and operational planning for children’s healthcare services.	CAN	General pediatrics	Develop a framework for defining and planning child and youth healthcare services across British Columbia, Canada.	A tiers of service framework was developed with each tier having a unique role in delivering services, clear delineation of responsibilities and requirements, and understanding relationships between tiers and interconnections of networks across the system. This framework builds on existing evidence to emphasize three areas: 1. Service areas including community- and hospital-based services; 2. Prevention; and 3. Health determinants as part of context of children’s health.	**6 tiers of service:** Tier 1: Prevention, primary & emergent health service Tier 2: General health service Tier 3: Child-focused health service Tier 4: Children’s comprehensive health service Tier 5: Children’s regional enhanced and subspecialty service Tier 6: Children’s provincial subspecialty service
(American Academy of Pediatrics et al., 2012)	Levels of neonatal care	USA	Maternal and newborn health	A revised policy statement reviewing current status of levels of newborn care definitions in United States. Neonatal levels of care are delineated by minimal capabilities, functional criteria, and provider types.	This revised statement updates designations to provide 1) a basis for comparison of health outcomes, resource use, and health care costs; 2) standardized nomenclature for public health; 3) uniform definitions for pediatricians and other health care professionals providing neonatal care; 4) a foundation for consistent standards of service by institutions, state health departments, and state, regional, and national organizations focused on improvement of perinatal care.	**4 levels of care** Level 1: Well newborn nursery providing neonatal resuscitation at every delivery, provide postnatal care to stable term newborns, stabilize newborn infants until transfer to higher level of care Level 2: Special care nursery to provide care for infants born >32 weeks gestation not requiring subspecialty services on an urgent basis, provide care for infants convalescing after intensive care Level 3: Neonatal intensive care units providing sustained life support, provide comprehensive care for infants born <32 weeks gestation. Provide prompt and readily available access to a full range of pediatric medical subspecialists including respiratory support, advanced imaging. Level 4: Regional neonatal intensive care units located within an institution with capability to provide surgical repair of complex congenital or acquired conditions, maintain a full range of pediatric medical subspecialists, pediatric surgical subspecialists, and pediatric anesthesiologists
(American College of Surgeons, 2015)	Optimal resources for children’s surgical care v.1.	USA	Subspecialty (pediatric Surgery)	Development of standards of children’s surgical center standards and expected scope of practice guidelines.	Standards of care guidelines include recommendations for defining optimal resource standards and matching them prospectively to children’s needs and designating scope of practice with appropriate resources at care facility.	**3 levels of care:** Level 1: Regional tertiary-care facility central to children’s health care system. Provide comprehensive care for all aspects of children’s surgical needs with adequate resources and subspecialty health teams, and research and medical education programs. Level 2: Surgical center expected to provide initial surgical care regardless of complexity and definitive care when appropriate. Stabilize patients with more complex needs until transfer to a higher care center. Level 3: Serves communities that do not have immediate access to level 1 or 2 centers. Provide prompt assessment, resuscitation, emergency operations, and stabilization until transfer to a higher care center.
(Frankel et al., 2019)	Criteria for critical care of infants and children: PICU admission, discharge, and triage practice statement and levels of care guidance	USA	PICU	Update to American Academy of Pediatrics and Society of Critical Care Medicine’s 2004 guidelines and levels of care for PICU.	Following a modified Delphi process, a panel of experts voted on recommendations of practice statements and levels of care for PICU. Development of practice statements and levels of care guidelines included important specifications for each PICU level of care including team structure, technology and equipment, education and training, quality metrics, admission and discharge criteria, and indications for transfer to a higher level of care.	**3 levels of PICU care:** Level 1: Community PICU provides a broad range of services and resources that may differ based on institution, hospital size, and referral base. Geographic setting will impact populations/ disease entities served, available providers and support services, relationships with other PICUs at various levels, and transport capabilities Level 2: Tertiary PICU provides advanced care for many medical and surgical illnesses in infants and children. Provide advanced ventilatory support, access to subspecialties, advanced technologies and services. Level 3: Quaternary specialized PICU provides regional care and serves large populations with large catchment areas. Provides comprehensive care to all complex patients, diagnosis-specific care, specialized equipment and subspecialty care teams. This highest level of PICU has readily available resources as a Children’s surgical center or trauma center.
(Kilpatrick et al., 2019)	Obstetric care consensus #9: Levels of maternal care	USA	Maternal and newborn health	To reaffirm uniform definitions, standardized descriptions of maternity facility capabilities and personnel and framework for addressing levels of maternal care. Reaffirm goals of levels of maternal care and clarify definitions, revise criteria.	Levels of maternal care are revised from original 2015 maternal care obstetric care Consensus to clarify terminology and include more recent data. To standardize a comprehensive system of perinatal regionalization and risk-appropriate maternal care, this classification system establishes levels of maternal care that pertain to basic care (level 1), specialty care (level 2), subspecialty care (level 3), and regional perinatal health care centers (level 4). Levels of maternal care provide definitions, capabilities, and health care providers for 4 levels of maternal care and for birth centers.	**4 levels of care:** Level 1: Basic care for low- to moderate-risk pregnancies with ability to detect, stabilize, and initiate management of unanticipated maternal-fetal or neonatal problems during antepartum, intrapartum, postpartum period until patient can be transferred to higher level of care Level 2: Specialty care for moderate- to high-risk antepartum, intrapartum, or postpartum conditions with specialized care teams Level 3: Subspecialty care for complex maternal medical conditions, obstetric complications, and fetal conditions with subspecialist care teams Level 4: Regional perinatal health care centers with on-site medical and surgical care of the most complex maternal conditions and critically ill pregnant women and fetuses throughout antepartum, intrapartum, and postpartum care
(National Health Service, 2016)	Pediatric critical care standards for London: level 1 and 2. London: national health service	UK	PICU	Review of standard of care for pediatric critical care level 1 and 2 within a district general hospital environment.	Standards of care are defined through three levels of pediatric critical care. Guidelines include establishing networks of critical care services, minimum quality standards and sharing of best practice, treatment at different developmental stages, and defining pediatric critical care interventions at locations with appropriate resources.	**3 levels of pediatric critical care**: Level 1: Basic critical care unit Level 2: Intermediate critical care unit in a hospital setting or tertiary setting Level 3: Advanced critical care unit
(Provincial Council for Maternal and Child Health, 2013)	Standardized maternal and newborn levels of care definitions.	CAN	Maternal and newborn health	Review of standard of care for maternal and newborn services.	Guidelines of care include services with care providers trained to provide maternal and newborn care (e.g., resuscitation and stabilization), a clearly established referral path to higher/ specialized levels of care, and established patient transfer protocols.	**3 levels of maternal and newborn care:** Level 1: Low-risk maternal and newborn care Level 2: Low-to-moderate maternal and newborn risk Level 3: High-risk maternal and newborn care with on-site surgical capability
(Provincial Council for Maternal and Child Health, 2021)	Pediatric levels of care	CAN	General pediatrics	Update standards of care defined by acuity, medical complexity, and procedural complexity to optimize access to care in geographically vast areas with limited expertise and resources.	Pediatric levels of care definitions are defined by low, medium, high acuity and medical complexity and procedural complexity when determining whether quality pediatric care can be safely provided. Three distinct levels of pediatric care are designated by the degree to which hospitals would be prepared to care for pediatric patients.	**3 levels of care:** Level 1: General care for pediatric patients, stabilized before being transferred to a higher level of care Level 2: Emergency care and urgent care and admit patients to a dedicated pediatric unit with a pediatrician. Care for pediatric patients with moderate acuity, complexity, and low procedural complexity Level 3: Care for patients with high acuity, high medical complexity, and high procedural complexity. Emergency departments dedicated to pediatric patients, including intensive care units.
(Rosenberg and Moss, 2004)	Guidelines and levels of care for pediatric intensive care units	USA	PICU	Review of scope of pediatric critical care services and elements of hospital care that are necessary to provide high-quality pediatric critical care.	Pediatric critical care is ideally provided by a PICU that meets level I multidisciplinary care for a wide range of complex, progressive, and rapidly changing medical, surgical, and traumatic disorders. There are minimum guidelines of care for PICU level I and level II including organization and administrative structure, physical facility, personnel, hospital facilities and services, drugs and equipment, prehospital care, quality improvement, training, and continuing education.	**2 levels of PICU care:** Level I: Provide care to most severely ill patient population, multidisciplinary care with specialized care of medical and surgical subspecialists Level II: Provide stabilization of critically ill children before transfer to another center or to avoid long-distance transfers for disorders of less complex or lower acuity. A level II PICU does not require a full spectrum of subspecialists. Level II units should be located according to documented demand or need and in concert with accepted principles of regionalization of medical care. Each level II unit must have a well-established communications system with a level I PICU.
(Tasmanian Government, 2018)	Tasmanian role delineation framework and clinical services profile version 4	AUS	General pediatrics	Review of clinical services and Tasmanian role delineation framework outlining standard of care for safe and sustainable services.	This framework will enable each clinical specialty to provide a safer patient transition along the full continuum of care and is a planning document to describe minimum support services, safety standards, skills and competencies, networking arrangements, and other service requirements necessary to provide a service at a specific level to ensure safe and appropriately supported clinical service delivery. Delineating level of clinical services, not the facilities themselves, consists of core clinical services and clinical support services.	**6 levels of service:** Level 1: Low complexity ambulatory care services Level 2: Low complexity inpatient and ambulatory care services Level 3: Low to moderate complexity inpatient and ambulatory care services Level 4: Moderate complexity inpatient and ambulatory care services Level 5: Moderate to high complexity inpatient and ambulatory care services Level 6: High complexity inpatient and ambulatory care services
(Child Health BC, 2018c)	Children’s medical services	CAN	General pediatric	Summary of tiers of service framework and approach for planning and coordinating children’s general health services in British Columbia, Canada.	Standards of care are defined within tiers of service levels and expected to align with service needs of children living within a geographic area of focus. Tiers of service are differentiated by patient level of acuity and medical complexity (i.e., low, medium, high). Responsibilities and requirements at each tier are divided into clinical services delivered by inpatient, hospital-based, outpatient, and community-based services.	**6 tiers of service:** Tier 1: Prevention, primary & emergent health service Tier 2: General health service Tier 3: Child-focused health service Tier 4: Children’s comprehensive health service Tier 5: Children’s regional enhanced & subspecialty health service Tier 6: Children’s provincial subspecialty health service
(Child Health BC, 2018b)	Children’s emergency medical services	CAN	Pediatric emergency	Summary of tiers of service framework and approach for planning and coordinating children’s emergency health services in British Columbia, Canada.	Standards of care are defined within tiers of service levels and provided to children within emergency department with acute and undifferentiated medical problems or injuries. Emergency department tiers of service was developed by interdisciplinary working group for services provided within a hospital.	**6 tiers of service:** Tier 1: Prevention, primary & emergent health service Tier 2: General health service Tier 3: Child-focused health service Tier 4: Children’s comprehensive health service Tier 5: Children’s regional enhanced & subspecialty health service Tier 6: Children’s provincial subspecialty health service
(Child Health BC, 2018a)	Children’s diabetes services	CAN	Subspecialty (diabetes)	Summary of tiers of service framework and approach for planning and coordinating children’s diabetes health services in British Columbia, Canada.	Standards of care are defined within tiers of service levels and include tier 1, 4, 5, and 6 for diabetes care. Responsibilities and requirements at each tier are summative. Service levels provided by a given team are expected to align with service needs of children living within the team’s geographic area of focus. Diabetes tiers of service provide a common language and methodology to describe responsibilities and requirements at each tier. Responsibilities and requirements at each tier are divided into clinical services delivered by outpatient and inpatient services.	**6 tiers of service:** Tier 1: Prevention, primary & emergent health service Tier 2: General health service Tier 3: Child-focused health service Tier 4: Children’s comprehensive health service Tier 5: Children’s regional enhanced & subspecialty health service Tier 6: Children’s provincial subspecialty health service
(Healthy Young Minds in Herts, 2023)	The CAMHS tier system explained	UK	Mental health	Summary of tiers of service framework for planning, commissioning, and delivering mental health services.	Hertfordshire’s emotional and wellbeing services for children and young people are commissioned through a tiered system.	**4 tiers of service**: Tier 1: Early intervention and prevention provided by schools, children’s centers, health visitors, school nurses, GPs, youth Connexions, helplines, and websites for support with external factors affecting wellbeing; with general emotional wellbeing; and for online therapeutic support and self-help. Tier 2: Early help and targeted services through community counseling, counseling or mentoring in schools, education psychologists, education support centers, targeted youth support teams, family support and free online counseling. Tier 3: Specialized mental health services, including eating disorder services. Services include specialist multidisciplinary approach to children and young people aged 0–19 who have a global learning disability and/or autistic spectrum disorder and their families. Tier 4: Inpatient provision and specialized day and inpatient units, where people with more severe mental health problems can be assessed and treated.
(New Zealand Ministry of Health, 2014)	The NZ role delineation model	NZ	General pediatric	Clinical services model to differentiate capability of services and complexity between services within, and across district health board providers.	Six levels of service complexity extend from community-based services through complex settings. This model requires a service to have an appropriate mix of subspecialties to achieve higher levels of complexity. This model creates specialty service capability levels that can be used to describe and understand patient services across the region.	**6 levels of service:** Level 1: Primary and community-based services provided by primary practitioners. Rural, provincial or urban setting Level 2: General and convalescent services, sometimes in rural communities, providing sub-acute care and access to acute services Level 3: Specialist services providing acute and elective care to communities Level 4: Specialized services with some subspecialization Level 5: Major specialist services with multiple subspecialties and subspecialty support Level 6: Supra specialist and definitive care services most complex of any subspecialty
(New Zealand Ministry of Health, 2011)	National plan for child Cancer services in New Zealand	NZ	Pediatric oncology	Role delineation model for pediatric oncology and hematology services.	Six levels of role delineation model differentiates between levels of complexity of services provided by New Zealand’s District Health Boards and enables services to be described consistently.	**6 levels of service:** Level 1: Primary and emergency care for stabilization before moving to appropriate higher level of service, no pediatric oncology services Level 2: General pediatric outpatient consultation before moving to appropriate higher level of service, no pediatric oncology services Level 3: Specialist pediatrician and health care providers with experience in pediatric oncology, supports outpatient pediatric chemotherapy, disease surveillance and late effects monitoring Level 4: Adult oncology service on site with enhanced laboratory and blood transfusion services and presence of radiation oncology Level 5: Pediatric subspecialty services available on site, supports all forms of cancer treatment, dedicated child/adolescent cancer unit, specialist oncologists and hematologists 24-h cover Level 6: Pediatric intensive care unit, dedicated child/adolescent palliative care, allogeneic hemopoietic stem cell transplantation
(New Zealand Ministry of Health, 2012)	Guidance for integrated pediatric palliative care services in New Zealand	NZ	Palliative care	Provide implementation-focused guidance to improve integration of palliative care service delivery to children and young people in New Zealand.	Develop guidance on levels of care that are evidence-based, provides analysis of current service delivery, reflects stakeholder involvement, proposes models of service delivery with a focus on implementation, can be implemented at no extra cost to health sector.	**3 levels of care:** Level 1: Palliative care approach are appropriately applied by all providers Level 2: General palliative care is provided by professionals with additional training and expertise at an intermediate level. Level 3: Specialist palliative care is provided by providers whose core activity is provision of palliative care services.
(Queensland Health, 2014)	Medical services― Children’s module overview	AUS	General pediatric	Summary of tiers of service framework for Queensland general pediatric services.	Guidelines for tiers of service framework include description of services provided, service requirements, workforce requirements, and risk considerations. There are required documented processes at each level of medical service with public or licensed private health facility for referral and transfer of patients to/from higher capability level services to provide safe, on-going management of children.	**6 tiers of service:** Tier 1: Ambulatory care Tier 2: Ambulatory care and limited inpatient service Tier 3: Inpatient services for single-system disorders and low-acuity medical conditions Tier 4: Inpatient services within a children’s ward Tier 5: Stand-alone multidisciplinary teams, specialties, rehabilitation, child protection and developmental services Tier 6: Complex medical services to children on statewide and interstate wide basis, specialist care teams
(Council, 2004)	CAMHS standard, national service framework for children, young people, and maternity services	UK	Mental health	Summary of tiers of service framework for planning, commissioning, and delivering mental health services.	Tiers of service framework ensures commissioning and availability of a comprehensive range of services to meet all mental health needs of children and young people in an area, with clear referral routes signposted between tiers.	**4 tiers of service**: Tier 1: Generalist services provided by practitioners who are not mental health specialists working in universal services; this includes GPs, health visitors, school nurses, teachers, social workers, youth justice workers and voluntary agencies. Tier 2: Practitioners at this level tend to be specialists working in community and primary care settings including primary mental health workers, psychologists and counselors Tier 3: Multidisciplinary team or service working in a community mental health clinic or child psychiatry outpatient service, providing a specialized service for children and young people with more severe, complex, and persistent disorders. Teams include child and adolescent psychiatrists, social workers, clinical psychologists, community psychiatric nurses, child psychotherapists, occupational therapists, art, music, and drama therapists. Tier 4: Essential tertiary level services for children and young people with most serious problems, such as day units, highly specialized outpatient teams and inpatient units. Specialists include secure forensic adolescent units, eating disorder units, and specialist neuro-psychiatric teams, usually serving more than one district or region.

Number of service levels are defined within three, four, and six levels. Services at level one provides primary and generalized health care in community and ambulatory settings, whereas services at levels three and six provide advanced and comprehensive critical care in regional/quaternary settings. For example, a framework of six tiers of service from Waibel et al. (2021) delineates minimum services each tier should be able to provide with higher tiers providing more complex and subspecialty services to larger regions and provincial populations. Tier one provides a wide breadth of general services accessible in most communities and tier six provides more comprehensive, sub-specialized services targeting complex pediatric health needs (Waibel et al., 2021). In contrast, specialty clinical services for Pediatric Intensive Care Unit (PICU) (Rosenberg and Moss, 2004) are defined by two levels of care, where level one manages most severely ill pediatric patients with subspecialty multidisciplinary care teams and level two stabilizes critically ill pediatric patients before transferring to a level one center. Differences between service levels and delineation of role and responsibilities of each level are specific to clinical service areas.

Most levels of service were reported by US (*n* = 8), Canada (*n* = 7) and UK (*n* = 6), with general pediatrics the most advanced (*n* = 6) reporting tiers of service (Table 3). Within US, standards of care for Women and Newborn Health are defined by four levels of care including a classification system of consistent definitions for neonatal care and organization of levels of maternal care by institution, state health department, regional and national organizations. Services are delineated within four levels of care by roles and responsibilities of healthcare providers delivering general, specialty, subspecialty care for complex conditions, and most complex and critically ill women and newborns (American Academy of Pediatrics et al., 2012; Barnea et al., 2021; Kilpatrick et al., 2019). Within Canada (British Columbia), standards of care for general pediatrics are defined by six tiers of service that define roles and responsibilities for delivering care according to patient level of acuity, medical complexity, and health centers with varying resources for inpatient, hospital-based, outpatient, and community-based services (Child Health BC, 2018c; Waibel et al., 2021). British Columbia’s six tiers of service framework was developed by synthesizing standards of pediatric service delivery and consulting provincial stakeholders to enhance definitions of community- and hospital-based service areas, health promotion strategies and prevention, and determinants of health contributing to children’s health (Waibel et al., 2021). Within UK, standards of care for pediatric and adolescent mental health are defined by four tiers of service, where a range of services are coordinated through referral for service between care settings and each tier of service (Sutton Council, 2004). Four tiers of service include early intervention in community sites and settings, targeted services through community counseling, specialized mental health services, and inpatient provision and specialized units for more severe mental health challenges to be assessed and treated.

## Integrated levels of service

Eight sources described integrated levels of service, with PICU (*n* = 2) and palliative care (n = 2) most advanced in defining their approach to integration (Table 4).

**Table 4.** Results of academic gray literature findings reporting pediatric integrated levels of service.

Author	Title	City	Clinical service area	Description of integrated levels of service
(Barnea et al., 2021)	From fragmented levels of care to integrated health care: Framework toward improved maternal and newborn health.	USA	Maternal and newborn health	An integrated model of care leverages a multidisciplinary team, including midwives, primary obstetricians, maternal-fetal medicine specialists, anesthetists, and other subspecialty clinicians to deliver a coordinated set of perinatal healthcare services based on the mother’s associated co-morbidities and level of risk.
(Boddy et al., 2013)	Standards for children’s surgery: Children’s forum	UK	Subspecialty (pediatric surgery)	Coordination of health provider roles and responsibilities across different levels of service to improve transitions in care and integration of care. A plan for service transfer may require formal service agreements.
(Brock et al., 2019)	Models of pediatric palliative oncology outpatient care‚ benefits, challenges, and opportunities.	USA	Pediatric oncology	Integrated care provides inpatient, outpatient, and home-based consultations to follow patients across hospitalizations and routine clinic visits. Teams coordinate patient care across settings to enhance continuity. Patients are referred to pediatric palliative care providers based on need, where an oncology team facilitates referrals and transfers across care settings.
(Darling et al., 2014)	Implementation of the Canadian Paediatric Society’s hyperbilirubinemia guidelines: A survey of Ontario hospitals	CAN	Maternal and newborn health	Care is coordinated and integrated through community-based care providers for newborns with pediatricians, family physicians, midwives, and nurse practitioners.
(National Health Service, 2016)	Pediatric critical care standards for London: level 1 and 2. London: National Health Service	UK	PICU	Three levels of service must be organized across hospital and community sites so that services are integrated for pediatric patients by a single group responsible for coordination and development of care pathways by a multidisciplinary team, nominated lead consultants for nurses, lead anesthetist and resuscitation officer.
(Rothschild and Derrington, 2020)	Palliative care for pediatric intensive care patients and families.	USA	Palliative care	Coordination and integration of care across specialist teams including transplant surgery, complex congenital heart disease, trauma, neurosurgery, and otorhinolaryngology.
(Rosenberg and Moss, 2004)	Guidelines and levels of care for pediatric intensive care units	USA	PICU	PICUs should be integrated with community-based services to ease transitions between hospitals or from the scene of an injury through integration with regional emergency health services. A standard written approach to emergencies involving emergency health services and PICUs should be formalized.
(New Zealand Ministry of Health, 2012)	Guidance for integrated pediatric palliative care services in New Zealand	NZ	Palliative care	Services are coordinated and integrated through provider networks. Care is integrated through specialist and generalist service delivery that collaborate through a district health board with coordinators that lead local needs assessment, data collection, and service development. Local specialist nursing staff should be integrated with community pediatric nursing teams to promote a model of integrated and comprehensive care delivered along a continuum, in a setting of choice, with respite and case management as central features.

**Figure 2.**
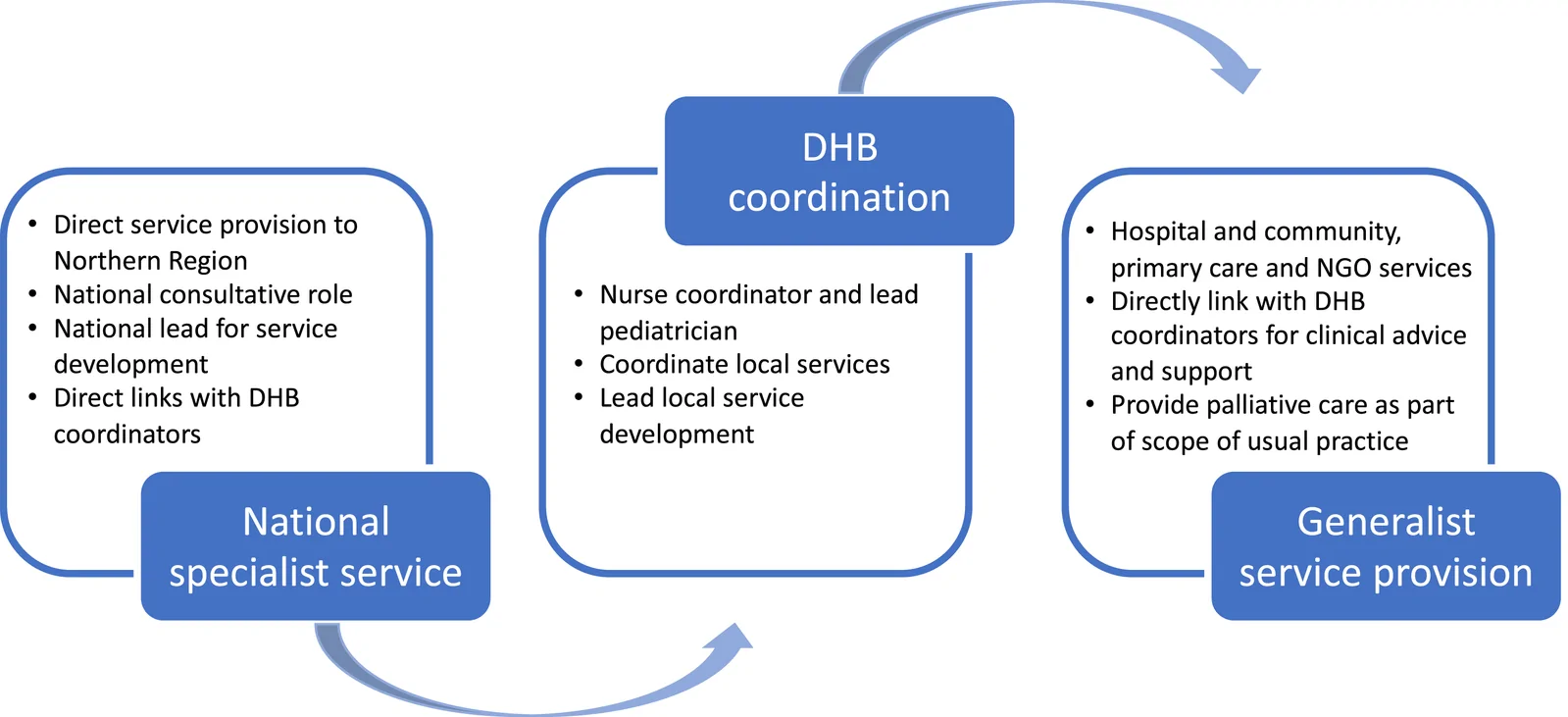
New Zealand pediatric palliative care system relationships. Source: New Zealand Ministry of Health. Guidance for integrated pediatric palliative care services in New Zealand [Internet]. 2012. Available from: https://www.health.govt.nz/system/files/documents/publications/guidance-integrated-paeiatric-palliative-care-services-nz.pdf.

All eight sources described integrated levels of care through integration of specialist and generalist services, including coordination of roles and responsibilities for health professionals across different levels of service, agreements for transferring patients between units or sites, and use of one electronic health record system across different levels of service. New Zealand’s framework for integrated pediatric palliative care was most advanced describing a comprehensive approach for organizing and planning integrated levels of service (New Zealand Ministry of Health, 2012). New Zealand’s framework (Figure 2) describes how national specialist services are coordinated by district health boards, responsible for organizing local generalist services within hospital, community, and primary care to provide palliative care as part of general scope of practice. Both national specialist and local generalist services are linked to district health boards where a nurse coordinator and lead pediatrician coordinate local services and service development.

## Discussion

This scoping review provides a comprehensive overview of current evidence on pediatric standards of care and levels of service, revealing critical themes that shape how care is structured and delivered across diverse settings. Thematic analysis highlighted consistent patterns in how service levels and standards of care are defined and linked to pediatric health outcomes. Importantly, the review uncovered significant geographical and contextual variations, reflecting how different countries approach pediatric service delivery based on their unique healthcare priorities. Of 53 sources identified, 27 sources reported on levels or tiers of service and eight described integrated levels or tiers of service. The US (*n* = 8), Canada (*n* = 7) and UK (*n* = 6) are most advanced with defining levels of service and New Zealand most advanced with describing integrated levels of pediatric palliative care. Almost all frameworks defined levels or tiers of service by increasing complexity, where higher levels or tiers provide more specialized services across larger regions and populations.

Pediatric levels of service frameworks provide important information for defining common language, practice guidelines, and planning and organizing services across jurisdictions, health care organizations, and levels of care. Levels of service frameworks we identified used a similar approach to defining levels of patient acuity and medical complexity, where each level is differentiated by roles and responsibilities of healthcare providers across care settings (e.g., community-based services, outpatient, hospital, and inpatient). Differences between country specific levels of service frameworks were defined by health system priorities such as, operationalizing roles and responsibilities of providers (Kilpatrick et al., 2019; Rosenberg and Moss, 2004), improving transitions between levels of care (Tasmanian Government, 2018), organizing and planning for services across urban and rural communities within local, regional, and state or provincial service areas (Waibel et al., 2021), and coordinating integrated services (New Zealand Ministry of Health, 2012). For example, Australia’s (Queensland and Tasmania) six tiers of service framework emphasize minimum service requirements to improve pediatric transitions across levels of care and health care settings (Tasmanian Government, 2018). Australia’s framework operationalizes roles and responsibilities of providers within each tier of clinical service to coordinate safe and continuous care management for patients moving to public or private health facilities (Queensland Health, 2014). Whereas New Zealand’s framework of three levels of service emphasizes a need for improving integrated standards of palliative care across different regions and health centers (New Zealand Ministry of Health, 2012). New Zealand operationalizes the role and responsibility of district health boards to coordinate and lead service development between nationalist specialist and generalist services by integrating palliative care services as usual scope of practice across all care settings (New Zealand Ministry of Health, 2012).

Few sources (n = 8) described integrated standards of care (Table 4), focusing on horizontal integration (coordinating care within same service levels) to reduce fragmentation and utilize multidisciplinary teams, as seen in perinatal services (Barnea et al., 2021). Vertical integration (coordinating different care levels) was described for PICU and community pathways (National Health Service, 2016), while clinical integration (continuity and coordination of care, disease management, and sharing of information) emphasized improving continuity and transitions between various care settings (Boddy et al., 2013; Brock et al., 2019; Darling et al., 2014; Rosenberg and Moss, 2004; Rothschild and Derrington, 2020). New Zealand’s integrated palliative care model exemplifies clinical and functional integration through district health boards, coordinating assessments, service development, and comprehensive care delivery (New Zealand Ministry of Health, 2012).

Integrated care frameworks are vital for coordinating pediatric services across fragmented systems, especially for pediatric patients and their families navigating specialist services and regions where urban hospitals manage rural hospitals (Cohen and Coller, 2020; Cumming, 2011; O’Shea et al., 2018). In Canada, proportions of Canadians living in rural areas vary greatly by province or territory. For example, in Atlantic provinces, most residents live in rural areas (Government of Canada, 2022), yet specialized pediatric services are centralized in Halifax, Nova Scotia. Defining roles, scopes, and responsibilities within an integrated framework could improve care navigation, reduce redundancies, and minimize delays (Splane et al., 2023). This review highlights gaps in describing and operationalizing integrated care standards, emphasizing a need for future research to refine these frameworks and clarify levels of service.

Levels of service frameworks clarify roles, responsibilities, and care standards across service levels, enabling consistent care delivery and alignment with patient needs (Rosenberg and Moss, 2004). Their practical implementation and adaptation to local contexts remains unclear (Shaw et al., 2014). We identified two case studies, from Canada and New Zealand, detailing health service planning approaches. In Canada, Waibel’s (2021) six-tier framework uses educational modules and self-assessments to enhance planning and coordination across care levels, fostering urban-rural partnerships and quality improvement. Utilizing this framework could enable stronger partnership between urban and rural sites, facilitate opportunities to learn from each other and increase understanding of rural versus urban health care contexts. These processes are particularly vital in developing countries (Carmone et al., 2020; Goma et al., 2017) where resource limitations and fragmented healthcare systems often hinder equitable service delivery. With this, improvements, innovation, and lessons learned can be shared beyond local contexts.

New Zealand is recognized for actively developing integrated approaches to pediatric services (Dempers and Gott, 2017; Garrett et al., 2020; Wodchis et al., 2015). Their 3-year phased approach for implementing pediatric palliative care provides guidance on which organizations are responsible for leading operationalization of integrated care and service development (New Zealand Ministry of Health, 2012). In year one, New Zealand established and resourced care networks, appointed and trained regional health board coordinators, developed guidelines, conducted needs assessments, and implemented after-hours support. Year two focused on disseminating guidelines, developing care coordination mechanisms, and establishing quality and research data systems. By year three, evaluation frameworks were created, and services were aligned with local Māori developments and evolving policies.

New Zealand’s approach to coordinating and implementing pediatric care exemplifies effective health system practice and sustainable change (Davies, 2006; Mackenzie et al., 2011). It integrates community networks, comprehensive provider training, and prioritizes quality improvement through needs assessments and service mapping. By using evaluation frameworks and aligning services with Māori developments, it delivers culturally responsive and evidence-based care. This strategy ensures equitable access, adaptability to policy changes, and resilience in service delivery (Wodchis et al., 2015), showcasing forward-thinking planning and collaboration to maintain high standards of care and address evolving health needs.

While these studies provide valuable insights into implementation, health system decision-makers and practitioners may benefit from using a systematic approach to guide adapting and implementing standards of care to their cultural and organizational context (Harrison et al., 2013). Drawing from implementation science literature, the three phase ADAPTE process (set-up, adaptation, and finalization) provides a systematic way for health organizations and jurisdictions to identify, critically appraise, and adapt standards of care guidelines for local use (ADAPTE Collaboration, 2009). The ADAPTE toolkit supports identifying, appraising, and adapting guidelines through three phases: preparation, adaptation, and finalization, ensuring relevance to local needs and policies (ADAPTE Collaboration, 2009). Frameworks like ADAPTE promote evidence-based decision-making, equitable resource allocation, and knowledge sharing across service levels (Bashiri et al., 2021; Fervers et al., 2011). In some cases, tertiary centers may have more resources dedicated to knowledge translation of best evidence. A levels model may accelerate knowledge translation to other sites with fewer pediatric admissions or high acuity low occurrence health conditions (Darzi et al., 2017). Standardized approaches similar to those outlined above can set the stage for comprehensive and fiscally responsible allocation of resources, services, and supports (Amer et al., 2015). From a health systems approach, this creates opportunity to share resources and knowledge, increasing capacity and quality of care provided at various levels.

Further research using similar implementation frameworks is needed to build the evidence base on *how* to operationalize and evaluate integrated pediatric levels of service frameworks in health systems. Researchers and health system partners should identify barriers and facilitators to implementing standards of care frameworks and develop tailored strategies to support their implementation. Further, evaluation efforts, including both process and outcome evaluations, are needed to examine impacts of these standards of care frameworks on achieving the quintuple aim of including improved patient, provider, health system, population, and health equity outcomes (Gerlach and Varcoe, 2021).

### Strengths and limitations

This rapid scoping review has several notable strengths. Rigor and quality were upheld by following established scoping review methodologies from PRISMA-ScR guidelines. A clearly defined protocol guided the review process, incorporating comprehensive search strategies across academic and gray literature, independent screening and data extraction by multiple reviewers, and thorough documentation to ensure transparency and consistency. There are also important limitations to consider. First, the review was limited to English-language publications, which may have excluded relevant evidence published in other languages. Second, the focus on high-income countries (Canada, US, UK, Australia, and New Zealand) limits the generalizability of findings to low- and middle-income contexts, where health system structures and resource constraints differ significantly. Finally, the search was restricted to publicly available sources, omitting internal or unpublished documents such as health organization policies and procedures, which may offer valuable insights into how pediatric service delivery frameworks are implemented in practice.

### Implications for practice

This review highlights the importance of adopting standardized pediatric levels of service frameworks to support consistent, equitable, and coordinated care delivery across diverse healthcare settings. Defining clear roles and responsibilities within these frameworks enables improved care planning, reduces fragmentation, and supports transitions between care levels, particularly vital for children with complex needs and for regions with urban-rural disparities in service access. Health system leaders and practitioners can look to models from countries like New Zealand, Australia, and Canada for practical strategies on operationalizing standards of care. Implementing evidence-based frameworks, such as the ADAPTE process, can guide local adaptation and foster collaboration across service levels, while also supporting culturally responsive care. As pediatric health systems face increasing demands, integrating these frameworks into practice offers a pathway toward more efficient, patient-centered, and sustainable service delivery.

## Conclusion

Pediatric standards of care and integrated levels of service frameworks provide important information for defining common language, practice guidelines, and planning and organizing services across jurisdictions, health care organizations, and levels of care. This review identified pediatric standards of care and integrated levels of service frameworks; however, health organizations would benefit from learning how other jurisdictions implement and adapt models of service delivery to local population needs and resources available. There is a need to strengthen processes for adapting pediatric standards of care to local contexts by engaging health system decision-makers, patients, and families as stakeholders impacted by guidelines. Further research is needed to build an evidence base of processes for operationalizing and implementing integrated services to advance ultimate goals of improving pediatric care that is comprehensive and coordinated across different service regions and levels of care.

## Supplemental Material

Supplemental Material - Standards of care for pediatric clinical service delivery: A rapid scoping review Supplemental Material for Standards of care for pediatric clinical service delivery: A rapid scoping review by Brittany Barber, Annette Elliott-Rose, Amanda Higgins, Kristy Hancock, Amy Mireault, Katie McDonald, Stacy Burgess, LeeAnn Larocque and Cassidy Christine in Journal of Child Health Care
