# Supplementary material for: Standards of care for pediatric clinical service delivery: A rapid scoping review
Source: J Child Health Care. 2025 Jul 3;30(3):527–64. doi: 10.1177/13674935251355056 (PMC13490659; doi:10.1177/13674935251355056)
Supplement: Supplementary file 1 [file sj-pdf-1-chc-10.1177_13674935251355056.pdf]

Supplementary Table I: Database Searches
All searches run on June 30, 2022

Database: CINAHL Full Text (EBSCO)

#	Query	Limiters/Expanders	Results
S1	(MH "Child+") OR (MH "Congenital, Hereditary, and Neonatal Diseases and Abnormalities") OR (MH "Adolescence+") OR (MH "Pediatrics+") OR (MH "Minors (Legal)")	Expanders - Apply related words; Apply equivalent subjects Search modes - Boolean/Phrase	1,084,256
S2	TI ((pediatric* or paediatric* or child* or newborn* or congenital* or infan* or baby or babies or neonat* or "pre-term" or preterm* or "premature birth*" or NICU or preschool* or "pre-school*" or schoolchild* or toddler* or boy or boys or girl* or pubescen* or juvenile* or teen* or youth* or "young adult*" or adolesc* or "pre-pubesc*" or prepubesc*)) OR AB ((pediatric* or paediatric* or child* or newborn* or congenital* or infan* or baby or babies or neonat* or "pre-term" or preterm* or "premature birth*" or NICU or preschool* or "pre-school*" or schoolchild* or toddler* or boy or boys or girl* or pubescen* or juvenile* or teen* or youth* or "young adult*" or adolesc* or "pre-pubesc*" or prepubesc*))	Expanders - Apply related words; Apply equivalent subjects Search modes - Boolean/Phrase	989,941
S3	SO (child* or adolesc* or pediat* or paediat*)	Expanders - Apply related words; Apply equivalent subjects Search modes - Boolean/Phrase	344,901
S4	S1 OR S2 OR S3	Expanders - Apply related words; Apply equivalent subjects Search modes - Boolean/Phrase	1,463,273
S5	TI (((standard# or level# or tier* or framework#) N2 (care or service# or health*))) OR AB (((standard# or level# or tier* or framework#) N2 (care or service# or health*)))	Expanders - Apply related words; Apply equivalent subjects Search modes - Boolean/Phrase	67,700

S6	(MH "Health Facility Planning+")	Expanders - Apply related words; Apply equivalent subjects Search modes - Boolean/Phrase	1,088
S7	S5 OR S6	Expanders - Apply related words; Apply equivalent subjects Search modes - Boolean/Phrase	68,772
S8	TI (((integrat* or coordinat* or comprehensive) N2 (care or service# or health*))) OR AB (((integrat* or coordinat* or comprehensive) N2 (care or service# or health*)))	Expanders - Apply related words; Apply equivalent subjects Search modes - Boolean/Phrase	49,400
S9	(MH "Health Care Delivery, Integrated")	Expanders - Apply related words; Apply equivalent subjects Search modes - Boolean/Phrase	13,662
S10	S8 OR S9	Expanders - Apply related words; Apply equivalent subjects Search modes - Boolean/Phrase	57,503
S11	S4 AND S7 AND S10	Expanders - Apply related words; Apply equivalent subjects Search modes - Boolean/Phrase	832

Database: Embase (Elsevier)

No.	Query	Results
1	'child'/exp OR 'newborn disease'/de OR 'infant'/exp OR 'adolescent'/exp OR 'pediatrics'/exp OR 'minor (person)'/de	4144656
2	pediatric*:ti,ab,kw OR paediatric*:ti,ab,kw OR child*:ti,ab,kw OR newborn*:ti,ab,kw OR congenital*:ti,ab,kw OR infan*:ti,ab,kw OR baby:ti,ab,kw OR babies:ti,ab,kw OR neonat*:ti,ab,kw OR 'pre-term':ti,ab,kw OR preterm*:ti,ab,kw OR 'premature birth*':ti,ab,kw OR nicu:ti,ab,kw OR preschool*:ti,ab,kw OR 'pre-school*':ti,ab,kw OR schoolchild*:ti,ab,kw OR	3853751

	toddler*:ti,ab,kw OR boy:ti,ab,kw OR boys:ti,ab,kw OR girl*:ti,ab,kw OR pubescen*:ti,ab,kw OR juvenile*:ti,ab,kw OR teen*:ti,ab,kw OR youth*:ti,ab,kw OR 'young adult*':ti,ab,kw OR adolesc*:ti,ab,kw OR 'pre-pubesc*':ti,ab,kw OR prepubesc*:ti,ab,kw	
3	child*:jt OR adolesc*:jt OR pediat*:jt OR paediat*:jt	1048355
4	#1 OR #2 OR #3	5529161
5	((standard$ OR level$ OR tier* OR framework$) NEAR/2 (care OR service$ OR health*)):ti,ab,kw	176092
6	'health care planning'/exp	109163
7	'professional standard'/exp	544510
8	#5 OR #6 OR #7	809990
9	((integrat* OR coordinat* OR comprehensive) NEAR/2 (care OR service$ OR health*)):ti,ab,kw	81945
10	'integrated health care system'/de	12683
11	#9 OR #10	89102
12	#4 AND #8 AND #11	2680

Database: Medline (Ovid)

#	Searches	Results
1	exp Child/ or "Congenital, Hereditary, and Neonatal Diseases and Abnormalities"/ or exp infant/ or adolescent/ or exp pediatrics/ or minors/	3882626
2	(pediatric* or paediatric* or child* or newborn* or congenital* or infan* or baby or babies or neonat* or pre-term or preterm* or premature birth* or NICU or preschool* or pre-school* or schoolchild* or toddler* or boy or boys or girl* or pubescen* or juvenile* or teen* or youth* or young adult* or adolesc* or pre-pubesc* or prepubesc*).ti,ab,kf.	2988982
3	(child* or adolesc* or pediat* or paediat*).jn.	285018
4	or/1-3	4870774
5	((standard? or level? or tier* or framework?) adj2 (care or service? or health*)).ti,ab,kf.	110261
6	exp Regional Health Planning/	40648
7	"Standard of Care"/	3527
8	or/5-7	151532
9	((integrat* or coordinat* or comprehensive) adj2 (care or service? or health*)).ti,ab,kf.	58596
10	"Delivery of Health Care, Integrated"/	13980
11	Comprehensive Health Care/	6756
12	or/9-11	72164
13	4 and 8 and 12	1035

Database: Scopus (Elsevier)

History Count	Search Terms	Results
1	TITLE-ABS-KEY (pediatric* OR paediatric* OR child* OR newborn* OR congenital* OR infan* OR baby OR babies OR neonat* OR "pre-term" OR preterm* OR "premature birth*" OR nicu OR preschool* OR "pre-school*" OR schoolchild* OR toddler* OR boy OR boys OR girl* OR pubescen* OR juvenile* OR teen* OR youth* OR "young adult*" OR adolesc* OR "pre-pubesc*" OR prepubesc*)	6,911,988 document results
2	SRCTITLE ((child* OR adolesc* OR pediat* OR paediat*))	1,063,166 document results
3	(TITLE-ABS-KEY (pediatric* OR paediatric* OR child* OR newborn* OR congenital* OR infan* OR baby OR babies OR neonat* OR "pre-term" OR preterm* OR "premature birth*" OR nicu OR preschool* OR "pre-school*" OR schoolchild* OR toddler* OR boy OR boys OR girl* OR pubescen* OR juvenile* OR teen* OR youth* OR "young adult*" OR adolesc* OR "pre-pubesc*" OR prepubesc*)) OR (SRCTITLE ((child* OR adolesc* OR pediat* OR paediat*)))	7,054,493 document results
4	TITLE-ABS-KEY ((standard* OR level* OR tier* OR framework*) W/2 (care OR service* OR health*))	247,613 document results
5	TITLE-ABS-KEY ((integrat* OR coordinat* OR comprehensive) W/2 (care OR service* OR health*))	142,596 document results
6	((TITLE-ABS-KEY (pediatric* OR paediatric* OR child* OR newborn* OR congenital* OR infan* OR baby OR babies OR neonat* OR "pre-term" OR preterm* OR "premature birth*" OR nicu OR preschool* OR "pre-school*" OR schoolchild* OR toddler* OR boy OR boys OR girl* OR pubescen* OR juvenile* OR teen* OR youth* OR "young adult*" OR adolesc* OR "pre-pubesc*" OR prepubesc*)) OR (SRCTITLE ((child* OR adolesc* OR pediat* OR paediat*)))) AND (TITLE-ABS-KEY ((standard* OR level* OR tier* OR framework*) W/2 (care OR service* OR health*))) AND (TITLE-ABS-KEY ((integrat* OR coordinat* OR comprehensive) W/2 (care OR service* OR health*)))	1,941 document results

Supplementary Table II: Advanced Google Searches

Google searches were conducted between September 20th to 27th, 2022

Country Filter	Search Terms	Results
Canada	1. (pediatric OR paediatric OR child OR children OR infant OR infants) AND (health OR healthcare) AND (standard OR standards OR level OR levels OR tier OR tiers OR tiered)	First 5 pages of results
	2. (youth OR "young adult" OR "young adults" OR "young person" OR "young people" OR adolescent OR adolescents) AND (health OR healthcare) AND (standard OR standards OR level OR levels OR tier OR tiers OR tiered)	
USA	1. (pediatric OR paediatric OR child OR children OR infant OR infants) AND (health OR healthcare) AND (standard OR standards OR level OR levels OR tier OR tiers OR tiered)	First 5 pages of results
	2. (youth OR "young adult" OR "young adults" OR "young person" OR "young people" OR adolescent OR adolescents) AND (health OR healthcare) AND (standard OR standards OR level OR levels OR tier OR tiers OR tiered)	
UK	1. (pediatric OR paediatric OR child OR children OR infant OR infants) AND (health OR healthcare) AND (standard OR standards OR level OR levels OR tier OR tiers OR tiered)	First 5 pages of results
	2. (youth OR "young adult" OR "young adults" OR "young person" OR "young people" OR adolescent OR adolescents) AND (health OR healthcare) AND (standard OR standards OR level OR levels OR tier OR tiers OR tiered)	
Australia	1. (pediatric OR paediatric OR child OR children OR infant OR infants) AND (health OR healthcare) AND (standard OR standards OR level OR levels OR tier OR tiers OR tiered)	First 5 pages of results
	2. (youth OR "young adult" OR "young adults" OR "young person" OR "young people" OR adolescent OR adolescents) AND (health OR healthcare) AND (standard OR standards OR level OR levels OR tier OR tiers OR tiered)	
New Zealand	1. (pediatric OR paediatric OR child OR children OR infant OR infants) AND (health OR healthcare) AND (standard OR standards OR level OR levels OR tier OR tiers OR tiered)	First 5 pages of results
	2. (youth OR "young adult" OR "young adults" OR "young person" OR "young people" OR adolescent OR adolescents) AND (health OR healthcare) AND (standard OR standards OR level OR levels OR tier OR tiers OR tiered)	

Supplementary Table III: Data Extraction Tool

<table>
<tr><th>Information to be Extracted</th><th colspan="2">Description</th></tr>
<tr><td>Date of Search</td><td colspan="2"></td></tr>
<tr><td>Country of origin</td><td colspan="2"></td></tr>
<tr><td>Year of publication</td><td colspan="2"></td></tr>
<tr><td>Author(s)</td><td colspan="2"></td></tr>
<tr><td>Study aim/purpose</td><td colspan="2"></td></tr>
<tr><td>Study population
(Children age, Diagnoses)</td><td colspan="2"></td></tr>
<tr><td>Pediatric clinical service area</td><td colspan="2"></td></tr>
<tr><td>Study setting
(Healthcare/clinical setting)</td><td colspan="2"></td></tr>
<tr><td>Qualitative Findings</td><td>Theme, pattern, concepts, categories</td><td>Illustrative quote</td></tr>
<tr><td>Guidelines/ Standards of care:

Reported Health System Outcomes:

Reported Patient Outcomes:

Implementation Strategy:
(barriers or facilitators)</td><td></td><td></td></tr>
<tr><td>Quantitative Findings:</td><td>Narrative description of findings</td><td>Findings (intervention group and control group, or before and after, if applicable)</td></tr>
<tr><td>Guidelines/ Standards of care:

Reported Health System Outcomes:

Reported Patient Outcomes:

Implementation Strategy:
(barriers or facilitators)</td><td></td><td></td></tr>
</table>

Supplementary Table IV: Results of academic and grey literature findings on pediatric standards of care

Academic Literature						
**Author**	**Title**	**Ctry**	**Clinical Service Area**	**Study Aim/ Purpose**	**Description of Standard or Guideline**	**Description of Levels or Tiers of Service**
(Adams et al., 2006)	Pharmacotherapy of lupus nephritis in children: a recommended treatment approach.	USA	Subspecialty Care (Nephrology)	Examine current standards of care, therapies in use, emerging treatments undergoing development and investigation, guidelines for treatment at the present time and suggestions for the comprehensive care of children with lupus nephritis.	Standard of care for highly active lupus nephritis in children remains intravenous cyclophosphamide, although preliminary experience suggests that the addition of rituximab may allow for remission induction with a reduction in cumulative cyclophosphamide dose. Combination therapies and newer agents appear promising for the future as our understanding of immune systems and dysregulation improves.	Not Available
(Barnea et al., 2021)	From fragmented levels of care to integrated health care: Framework toward improved maternal and newborn health.	USA	Women and Newborn Health	Develop an integrated tiered model of care that leverages a multidisciplinary team to deliver a coordinated set of perinatal healthcare services based on the mother's associated co-morbidities and level of risk.	The recommended standard of care for integrated maternal care includes timely access to care and counseling, coordinated care for pregnancy and postpartum, protocols for on-going assessment and transfer to high level care, and hospital level of care based on trained personnel. Resources for maternal care at the point of entry into care enables risk assessment (e.g., low, moderate, and high risk) and a plan for integrated care to start right away. Timely risk assessment minimizes maternal-newborn dyad outcome.	**4 Levels of Obstetric Care**:<br>Level I: Low-risk uncomplicated pregnancy, well-controlled gestational diabetes, mild pre-eclampsia, twins<br>Level II: Level I plus additional risk factors like complex cesarian delivery, medical complications requiring close monitoring (i.e., asthma, chronic hypertension, diabetes)<br>Level III: Level II plus additional factors like maternal complications (cardiac, coagulation disorders), immune disorders, placental pathologies (accrete, previa, bleeding), severe preterm pre-eclampsia<br>Level IV: Level III plus additional factors like severe maternal disease (cardiac, pulmonary), maternal disease requiring complex surgery, unstable maternal condition.
(Bhatnagar and Hall, 2018)	Major bleeding disorders: Diagnosis, classification, management, and recent developments in haemophilia	UK	Subspecialty (Haematology)	Review standards of care for children in the UK with the most common major bleeding disorder, haemophilia, and new developments in therapy.	Prophylaxis is the standard of care for children with severe haemophilia. Recombinant factor replacement therapy is a secondary standard of care for haemophilia treatment in the UK. Recent treatment developments include human cell line recombinant FVIII products, extended half-life recombinant factor concentrates, and PD-FVIII factor concentrates.	Not Available

Supplementary Table IV: Results of academic and grey literature findings on pediatric standards of care *(continued)*

Author	Title	Ctry	Clinical Service Area	Study Aim/ Purpose	Description of Standard or Guideline	Description of Levels or Tiers of Service
(Brock et al., 2019)	Models of Pediatric Palliative Oncology Outpatient Care, Benefits, Challenges, and Opportunities.	USA	Pediatric Oncology	Synthesize five institutions' unique outpatient pediatric palliative oncology model with their respective benefits and challenges.	The optimal planning process for delivering outpatient pediatric palliative care will delineate available resources and identify the ideal model for an institution, be it an embedded, standalone, floating, consultative, and/or telemedicine model. An assessment of patient, provider, and system needs can aid the selection of the best-fit model and desired patient population, communication preferences, note templates, frequency of follow-up, and choice of clinic name. Standards of care are described under five different pediatric oncology models including 1. floating clinic, 2. disease-specific embedded team, 3. trigger-based clinic using oncology clinic space (embedded), 4. consultation-based clinic using oncology clinic space, 5. telehealth clinic.	Not Available
(Clarkson et al., 2005)	Alveolar bone grafting: Achieving the organisational standards determined by CSAG, a baseline audit at the Birmingham Children's Hospital	UK	Subspecialty (Plastic Surgery)	Retrospective clinical process audit of the alveolar bone graft success rate and changes required for improving clinical outcomes.	Recommended protocol for standard of care for radiological assessment, centralisation of the service, and prospective data collection.<br>1. Aged 8 years: assessment with Surgeon/Dentist/Orthodontist<br>2. Start of orthodontic treatment: Surgery within 3 months<br>3. Postoperative review with surgeon within 2 weeks<br>4. 6 months after graft: Follow-up review with surgeon and orthodontist<br>5. After canine fully erupted: Revert to cleft protocol pathway	Not Available
(Darling et al., 2014)	Implementation of the Canadian Paediatric Society's hyperbilirubinemia guidelines: A survey of Ontario hospitals	CAN	Women and Newborn Health	Examine whether hospitals implemented neonatal standard of care guidelines and investigate how guideline recommended care is organized, including factors influencing guideline implementation.	Recommended standard of care include universal neonatal bilirubin screening, timely follow-up for babies requiring repeat bilirubin testing after hospital discharge, and follow-up from community-based care providers including paediatricians, family physicians, midwives, and nurse practitioners.	**3 Levels of Neonatal Care**:<br>Level 1: Basic level of service<br>Level 2: Intermediate care<br>Level 3: Specialized level of service

Supplementary Table IV: Results of academic and grey literature findings on pediatric standards of care *(continued)*

Author	Title	Ctry	Clinical Service Area	Study Aim/ Purpose	Description of Standard or Guideline	Description of Levels or Tiers of Service
(Davies, 2006)	The potential of integrated multi-agency care pathways for children.	UK	Palliative Care	Review of the Framework for the Development of Integrated Multi-agency Care Pathways for Children with Life-Threatening and Life Limiting Conditions, and its potential to transform the delivery of palliative care services.	The integrated multi-agency care pathway includes three stages.<br>1. Diagnosis or recognition of a life-threatening or life-limiting condition: disclosure of every child's prognosis in a face-to-face discussion with information provided both for the child and family and agreed transfer plan involving hospital, community services and the family with resources coordinated before leaving hospital.<br>2. Living with a life-threatening or life-limiting condition: every family should receive a multi-agency assessment of their needs as soon as possible after diagnosis with a multi-agency care plan agreed with them for delivery of coordinated care and support to meet their individual needs.<br>3. End of life and bereavement: every child and family should be helped to decide on an end-of-life plan and should be provided with care and support to achieve this as closely as possible.	Not Available
(Ebbels et al., 2017)	Evidence-based pathways to intervention for children with language disorders	UK	Subspecialty (Language Difficulties and Disorders)	Examine effectiveness of interventions for children with language disorders across different tiers and propose an evidence-based model of speech and language therapy service delivery to aid clinical decision-making.	A Tiered approach to service delivery is currently recommended, whereby services become increasingly specialised and individualised for children with greater needs. Paediatric speech and language therapist roles often involve planning individualised interventions for specific children and contribute to delivery of care at each Tier 1, 2, and 3 of services.	**3 Tiers of Service:**<br>Tier 1: Universal services with high-quality teaching<br>Tier 2: Targeted services providing education-led language programmes<br>Tier 3: Specialist services providing individualized interventions delivered by speech and language therapists

Supplementary Table IV: Results of academic and grey literature findings on pediatric standards of care *(continued)*

Author	Title	Ctry	Clinical Service Area	Study Aim/ Purpose	Description of Standard or Guideline	Description of Levels or Tiers of Service
(Evans, 2018)	Setting the standard for care of children with long-term conditions.	UK	General Pediatrics	Report of standards of care for children with ongoing health needs.	11 Standards of care include:<br>1. Referrals are responded to within 5 days<br>2. Single point of contact available within 3 days<br>3. Information services available<br>4. Pediatricians have 24/7 access for urgent advice from specialties<br>5. Non-urgent clinical communication sent to family practice within 5 days<br>6. Dedicated professional handle transitional care<br>7. Electronic shared healthcare records<br>8. Mental health services available for psychological needs<br>9. Dedicated lead for children at executive board level<br>10. Co-design of child services with involvement of parents/ carers<br>11. System to monitor, review and improe integration of local child health services	Not Available
(Fath, 2008)	Common denominators in standards	USA	Subspecialty (Pediatric Nursing)	Compare content and intent of standards of care in pediatric nursing from American Nurses Association and Hospital Accreditation Standards.	There are 6 standards of pediatric nursing care including:<br>1. Assessment: Collection of comprehensive health data, involving the child/family and other healthcare providers.<br>2. Diagnosis: Analysis and synthesis of data collected and diagnostic tests to determine diagnoses.<br>3. Outcome Identification: Identify expected outcomes individualized to the child and the family. Outcomes can be defined as goals or expected changes in the health status of the child. Outcomes are measurable, observable behaviors.<br>4. Planning: Healthcare providers develop a plan of care outlining interventions to attain expected outcomes. The interdisciplinary plan describes care to be delivered across a continuum.<br>5. Implementation: Healthcare providers implement the interventions identified in the established plan of care.<br>6. Evaluation: The pediatric nurse determines the child's and family's progress toward attainment of expected outcomes and the effectiveness of nursing care provided.	Not Available

Supplementary Table IV: Results of academic and grey literature findings on pediatric standards of care *(continued)*

Author	Title	Ctry	Clinical Service Area	Study Aim/ Purpose	Description of Standard or Guideline	Description of Levels or Tiers of Service
(Finella et al., 2007)	IMPaCCT. Standard pediatric palliative care in Europe	UK	Palliative Care	Compare paediatric palliative care, best practices, and minimum standards agreed. IMPaCCT recommends these standards become implemented in all European countries.	IMPaCCT recommends the following minimum core standards of care for Europe. 1. Provision of care, 2. Unit of Care, 3. Care Team, 4. Care co-ordinator/Key worker, 5. Symptom management, 6. Respite Care, 7. Bereavement, 8. Age-appropriate care, 9. Education and training 10. Funding for palliative services 11. Euthanasia, 12. Symptom recognition and assessment, 13. Core principles of symptom management, 14. Core principles of pain management	**3 Levels of Paediatric Palliative Care:** Level 1: A palliative care approach. Palliative care principles should be appropriately applied by all healthcare professionals Level 2: General palliative care. At an intermediate level, a proportion of patients and families will benefit from the expertise of healthcare professionals who, although not engaged full time in palliative care, have had some additional training and experience in palliative care. Level 3: Specialist palliative care. Specialist palliative care services are those whose core activity is the provision of palliative care.
(Gauger et al., 2018)	Integrative and collaborative care models between pediatric oral health and primary care providers: a scoping review of the literature.	USA	Dentistry	Review evaluating different types of care models that exist between oral health and primary care providers in pediatric settings.	Integrative and collaborative care models are important for increasing oral health practices and referrals to a range of oral health services. Levels of collaboration are described through three levels including: Low (e.g., off site referral for comprehensive care), Medium (e.g., minor on site procedures), and High (e.g., comprehensive care provided on site).	Not Available
(Glomb et al., 2017)	Prioritising minimum standards of emergency care for children in resource-limited settings	USA	Pediatric Emergency	Identify reasonable minimum standards to provide safe and effective care for acutely ill children in resource-limited settings.	Minimum standards of care from International Federation of Emergency Medicine remain unrealistic for many developing nations. A list of 145 minimum standards of care were used to identify 28 variables as "must have immediately" by panel of physicians in resource limited settings including personnel (n=7), equipment (n=2), and policies (n=19).	Not Available
(Hayles et al., 2015)	Delivering healthcare services to children with cerebral palsy and their families: a narrative review	AUS	Subspecialty (cerebral palsy)	Narrative review to identify the evidence for current approaches to healthcare service delivery for children with cerebral palsy.	Seven guidelines or standards of care for children with cerebral palsy and their families were identified. These consisted of three guidelines or standards developed by an individual hospital or state department of health in the USA, one guide published by a humanitarian organisation, two reference documents published within physician reference books and one peer-reviewed journal article. Each guideline or standard of care provides both general and specific information for the care of children with cerebral palsy, with the information organised by age group, body system, or potential presenting problems.	Not Available

Supplementary Table IV: Results of academic and grey literature findings on pediatric standards of care *(continued)*

Author	Title	Ctry	Clinical Service Area	Study Aim/ Purpose	Description of Standard or Guideline	Description of Levels or Tiers of Service
(Hsu et al., 2019)	Executive Summary: Criteria for Critical Care of Infants and Children: PICU Admission, Discharge, and Triage Practice Statement and Levels of Care Guidance.	USA	PICU	Summary of revised guidelines and levels of PICU care that will enable hospitals, institutions, and individuals to develop the appropriate PICU for their community needs.	An update of the American Academy of Pediatrics and Society of Critical Care Medicine's 2004 Guidelines and levels of care for PICUs. Specific recommendations include 1. PICU level of care admission criteria, 2. ICU structure and provider staffing model, 3. ICU personnel and resources, 4. Performance improvement and patient safety, 5. Equipment and technology, 6. PICU discharge and transfer criteria.	**3 Levels of PICU Care:**<br>1. Community based PICU: centers providing Level II PICU care with a broad range of services and resources and located in general medical-surgical institutions with capability of treating pediatric patients<br>2. Tertiary PICU: centers providing Level II advanced care for many medical and surgical illnesses in infants and children<br>3. Quaternary or specialized PICU: facility providing regional comprehensive care and serving large populations with complex patients. Highest level PICU facility supporting Level I and Level II centers.
(Maynard and Lynn, 2016a)	Development of a logic model to support a network approach in delivering 24/7 children's palliative care: part one...first of a two-part article	UK	Palliative Care	The first of a two-part article aiming to evaluate a 24/7 symptom-management service for children with life-limiting conditions and a model for palliative care nursing.	Guidelines of palliative care include: 1. 24/7 access to high-quality symptom management for all children and young people that need it, 2. Provide children and families with choices about location of care, 3. Contribute to the development of a medical support network, 4. Work in partnership with statutory services, 5. Act as a catalyst for service improvement, learning and development, 5. Find out 'what works' for children, families, and professionals.	Not Available
(Maynard and Lynn, 2016b)	Development of a logic model to support a network approach in delivering 24/7 children's palliative care: part two.	UK	Palliative Care	The second of a two-part article aiming to develop and evaluate a 24/7 symptom-management service for children with life-limiting conditions and a model for palliative care nursing.	Guidelines for delivering palliative care requires distribution of caseload and categorization of disorder groups across a variety of localities, educational establishments, acute and community health care environments. Diagnostic categories include:<br>Group 1. Life-threatening conditions for which curative treatment may be feasible but can fail,<br>Group 2. Conditions where premature death is inevitable,<br>Group 3. Progressive conditions without curative treatment options,<br>Group 4. Irreversible but non-progressive conditions causing severe disability, leading to susceptibility to health complications and likelihood of premature death	Not Available

Supplementary Table IV: Results of academic and grey literature findings on pediatric standards of care *(continued)*

Author	Title	Ctry	Clinical Service Area	Study Aim/ Purpose	Description of Standard or Guideline	Description of Levels or Tiers of Service
(Ogle et al., 2018)	Levels of type 1 diabetes care in children and adolescents for countries at varying resource levels.	AUS	Subspecialty (Diabetes)	Develop a levels of care framework for young people with Type 1 diabetes to help facilitate the work of policy makers, health planners, professional and lay advocates, in countries where comprehensive guideline-based care is mostly inaccessible.	Guidelines of care include services delivered within tiers including minimal, standard, and comprehensive care. Within each level details on insulin therapy, blood glucose monitoring, HbA1c testing, complications screening and diabetes education and multi-disciplinary team care are provided.	**3 Tiers of Service:**<br>Tier 1: Lowest level, designated 1A, human insulin (if available), is given through two injections daily.<br>Tier 2: Intermediate care is multiple daily injections ("basal bolus regimen"), accompanied by HbA1c testing and diabetes education provided by a pediatric or adult endocrinologist and/or a diabetes nurse educator.<br>Tier 3: Comprehensive, guidelines-based care.
(Pordes et al., 2018)	Models of care delivery for children with medical complexity	CAN	Subspecialty (Chronic Conditions)	Examine current models of care addressing multifaceted needs of Children with medical complexity and their families.	Standards of care delivery with children with medical complexity include:<br>1. Primary care-centered models (PCC): Community or tertiary care with emphasis on services that are continuous, coordinated, compassionate, and culturally appropriate<br>2. Consultative or co-management-centered models (CC): Providers in subspecialty programs, or more general complex care programs at tertiary care centers, deliver care coordination services<br>3. Episode-based models (EB): Time or location-limited interventions focused on providing medical management and care coordination for a specific illness episode or transitional period	Not Available
(Rogers, 2020)	Developing a community-based integrated children's bladder and bowel service.	UK	Subspecialty (Bladder and bowel)	Develop an integrated children's bladder and bowel service to ensure affected children are seen at the right time and place and by the right person.	Guidelines for delivering children's bladder and bowel care should focus on appropriate level of service (level 1 and 2) and integrated services between general health services and community-based care to improve self-management of care.	**2 Levels of Care:**<br>Level 1: Universal services provided by school nurses in the early identification and first-line management of bladder and bowel problems, such as delayed toilet training, bedwetting, and the onset of constipation.<br>Level 2: Nurse-led, community- based, children's bladder and bowel (continence) service. Comprehensive bladder and bowel assessments and treatment for children and youth from 0–19 years with bladder and/or bowel problems, and/or delayed toilet training, where universal level 1 interventions have not resolved the problem.

Supplementary Table IV: Results of academic and grey literature findings on pediatric standards of care *(continued)*

Author	Title	Ctry	Clinical Service Area	Study Aim/ Purpose	Description of Standard or Guideline	Description of Levels or Tiers of Service
(Rothschild and Derrington, 2020)	Palliative care for pediatric intensive care patients and families.	USA	Palliative Care	Review of evidence and best practices for integrating palliative care into the pediatric intensive care unit (PICU) with a focus on surgical patients.	Palliative care is best integrated in a tiered approach with primary palliative care provided by PICU and surgical providers including basic symptom management, high-quality communication, and end-of-life care. Secondary and tertiary levels of care involve unit or team-based champions with subspecialty care teams.	**3 Levels of Service:**<br>1. Primary Palliative Care: provided by members of primary teams, considered part of core competencies for the field.<br>2. Secondary Palliative Care: provided by "champions" within primary team who have additional expertise and/or training in palliative care principles, champions collaborate often with subspecialty palliative care team<br>3. Tertiary Palliative Care: provided by the subspecialty palliative care team
(Scialla et al., 2018)	Delivery of care consistent with the psychosocial standards in pediatric cancer: Current practices in the United States.	USA	Pediatric Oncology	Review of pediatric oncology treatment and factors contributing to perceptions of the quality of psychosocial care provided.	Standards of care for diagnosis and treatment phases include:<br>PSS1: Assessment of psychosocial needs<br>PSS2: Monitoring of neurocognitive problems<br>PSS3: Screening in long-term survivorship<br>PSS4: Psychosocial support and intervention<br>PSS5: Assessment of financial need (at diagnosis & ongoing)<br>PSS5: Assessment factors ... access to care (initial & ongoing)<br>PSS6: Parental mental health<br>PSS7: Psychoeducation, information, anticipatory guidance<br>PSS8: Preparatory information about invasive procedures and psychosocial care for invasive procedures<br>PSS9: Opportunities for social interactions<br>PSS10: Psychosocial support and interventions for siblings<br>PSS11: Support for school re-entry<br>PSS12: Adherence to treatment is assessed and monitored<br>PSS13: Palliative care concepts throughout disease process and developmentally appropriate end of life care<br>PSS14: Psychosocial care after a child's death	Not Available

Supplementary Table IV: Results of academic and grey literature findings on pediatric standards of care *(continued)*

Author	Title	Ctry	Clinical Service Area	Study Aim/ Purpose	Description of Standard or Guideline	Description of Levels or Tiers of Service
(Spencer et al., 2019)	Implementation of Off-Site Integrated Care for Children: A Scoping Review	USA	Mental Health	Review four models of off-site pediatric integrated mental health care to identify outcomes and factors that influence implementation.	Models of off-site integration for mental health services include<br>1. Direct in-person (in-person evaluations at specialty agencies with coordination through primary care)<br>2. Direct remote (telepsychiatry)<br>3. Indirect in-person (when primary care providers convene in person for case conferences)<br>4. Indirect remote (telephone psychiatry consultation programs to primary care providers)	Not Available
(Waibel et al., 2021)	Development of the Tiers of Service framework to support system and operational planning for children's healthcare services.	CAN	General Pediatrics	Develop a framework for defining and planning child and youth healthcare services across British Columbia, Canada.	A Tiers of Service framework was developed with each tier having a unique role in delivering services, clear delineation of responsibilities and requirements, and understanding relationships between tiers and interconnections of networks across the system. The developed framework builds on existing evidence to emphasize three areas: 1. Service areas including community- and hospital-based services; 2. Prevention; and 3. Health determinants as part of context of children's health.	**6 Tiers of Service:**<br>Tier 1: Prevention, primary & emergent health service<br>Tier 2: General health service<br>Tier 3: Child-focused health service<br>Tier 4: Children's comprehensive health service<br>Tier 5: Children's regional enhanced and subspecialty service<br>Tier 6: Children's provincial subspecialty service
(Wiener, Viola, et al., 2015)	Pediatric psycho-oncology care: standards, guidelines, and consensus reports.	USA	Pediatric Oncology	Identify existing guidelines, standards, or consensus-based reports for psychosocial care of children with cancer and their families.	Five published standards of care exist from American-based organizations, Canadian Association of Pediatric Oncology, International Society of Pediatric Oncology, and the European Society of Paediatric Oncology. Several published guidelines identified "ideal" psychosocial care for a child with cancer including recommendations:<br>1. Comprehensive pediatric hematology/oncology program to be implemented throughout the disease trajectory,<br>2. Psychosocial needs of cancer patients of all ages and described consequences of unmet needs, and<br>3. Implementation of standards at a national level to improve Quality-of-Care for Children with cancer, increasing survival rates and enhancing the Quality-of-Life for childhood cancer survivors.	Not Available

Supplementary Table IV: Results of academic and grey literature findings on pediatric standards of care *(continued)*

Author	Title	Ctry	Clinical Service Area	Study Aim/ Purpose	Description of Standard or Guideline	Description of Levels or Tiers of Service
(Woodland et al., 2010)	Health service delivery for newly arrived refugee children: A framework for good practice	AUS	Subspeciality (Refugee Care)	Propose a framework for good practice to promote improved access, equity and quality of care in service delivery for newly arrived refugee children.	Comprehensive health care for refugee children is provided through three models of care including; 1. General Practitioners in private practice with support from public health services, 2. Specialized paediatric screening and/ or referral clinics in hospital settings, 3. Specialized family screening clinics in hospital or community settings. Guidelines for good practice in health service delivery with refugee children include 10 elements. 1. Routine comprehensive health screening, 2. Coordination of initial and ongoing health care, 3. Integration of physical, developmental, and psychological health care, 4. Consumer participation, 5. Culturally and linguistically appropriate service provision, 6. Inter-sectoral collaboration, 7. Accessible and affordable services and treatments, 8. Data collection and evaluation to inform evidence-based practice, 9. Capacity building and sustainability, 10. Advocacy.	Not Available
(American Academy of Pediatrics et al., 2012)	Levels of Neonatal Care	USA	Women and Newborn Health	A revised policy statement reviewing the current status of levels of newborn care definitions in the United States. Neonatal levels of care are delineated by minimal capabilities, functional criteria, and provider types.	This revised statement updates designations to provide 1) a basis for comparison of health outcomes, resource use, and health care costs; 2) standardized nomenclature for public health; 3) uniform definitions for pediatricians and other health care professionals providing neonatal care; 4) a foundation for consistent standards of service by institutions, state health departments, and state, regional, and national organizations focused on improvement of perinatal care.	**4 Levels of Care**<br>Level 1: Well newborn nursery providing neonatal resuscitation at every delivery, provide postnatal care to stable term newborns, stabilize newborn infants until transfer to higher level of care<br>Level 2: Special care nursery to provide care for infants born > 32 weeks gestation not requiring subspecialty services on an urgent basis, provide care for infants convalescing after intensive care<br>Level 3: Neonatal intensive care units providing sustained life support, provide comprehensive care for infants born <32 weeks gestation. Provide prompt and readily available access to a full range of pediatric medical subspecialists including respiratory support, advanced imaging.<br>Level 4: Regional neonatal intensive care units located within an institution with the capability to provide surgical repair of complex congenital or acquired conditions, maintain a full range of pediatric medical subspecialists, pediatric surgical subspecialists, and pediatric anesthesiologists.

Supplementary Table IV: Results of academic and grey literature findings on pediatric standards of care *(continued)*

Author	Title	Ctry	Clinical Service Area	Study Aim/ Purpose	Description of Standard or Guideline	Description of Levels or Tiers of Service
(American College of Surgeons, 2015)	Optimal resources for children's surgical care v.1.	USA	Subspeciality (Pediatric Surgery)	Development of standards of children's surgical center standards and expected scope of practice guidelines.	Standards of care guidelines include recommendations for defining optimal resource standards and matching them prospectively to children's needs and designating scope of practice with appropriate resources at care facility.	**3 Levels of Care:**<br>Level 1: Regional tertiary-care facility central to children's health care system. Provide comprehensive care for all aspects of children's surgical needs with adequate resources and subspecialty health teams, and research and medical education programs.<br>Level 2: Surgical center expected to provide initial surgical care regardless of complexity and definitive care when appropriate. Stabilize patients with more complex needs until transfer to a higher care center.<br>Level 3: Serves communities that do not have immediate access to Level 1 or 2 centers. Provide prompt assessment, resuscitation, emergency operations, and stabilization until transfer to a higher care center.
(Boddy et al., 2013)	Standards for Children's Surgery: Children's Forum	UK	Subspecialty (Pediatric Surgery)	Summary of standards of care for children's surgery.	Standards of surgery care include:<br>1. Configuration of Services: Surgical services should be designed and delivered as part of a resourced network with a clinical lead.<br>2. Governance and Leadership: Commitment from the executive team and senior staff to provision high quality children's surgical service, with a multidisciplinary children's surgery committee reporting to the board.<br>3. Education and Training: pediatric clinical activity and training to maintain minimum competencies and consistent with their position.<br>4. Patients and Families: System of communicating with parents and families with a dedicated member of staff throughout admission<br>5. Delivery and Environment of Care: Treating children in safe, suitably staffed and equipped environments.	Not Available

Supplementary Table IV: Results of academic and grey literature findings on pediatric standards of care *(continued)*

Author	Title	Ctry	Clinical Service Area	Study Aim/ Purpose	Description of Standard or Guideline	Description of Levels or Tiers of Service
(Falster et al., 2012)	Development of a maternity hospital classification for use in perinatal research	AUS	Women and Newborn Health	Develop a maternity hospital classification with 13 obstetric groupings, based on neonatal care capability, urban and rural location, annual average number of births and public/ private hospital status.	The creation of a new maternity hospital classification was based on 4 dimensions of service level: neonatal care capability (tertiary NICU; non tertiary public, continuous positive airways pressure facilities, trained staff); geography (urban and rural location); annual average number of births (>1000, 500-999, 20-499 and <20 births); and hospital status (public or private). A case study of early elective births demonstrate that neonatal morbidity differs according to maternity hospital classification, and the classification of 13 obstetric grouping can be collapsed into subsets to support clinical and policy decision-making practices.	Not Available
(Frankel et al., 2019)	Criteria for critical care of infants and children: PICU admission, discharge, and triage practice statement and levels of care guidance	USA	PICU	Update to the American Academy of Pediatrics and Society of Critical Care Medicine's 2004 Guidelines and levels of care for PICU.	Following an modified Delphi process, a panel of experts voted on recommendations of practice statements and levels of care for PICU. Development of practice statements and levels of care guidelines included important specifications for each PICU level of care including team structure, technology and equipment, education and training, quality metrics, admission and discharge criteria, and indications for transfer to a higher level of care.	**3 Levels of PICU Care:**<br>Community PICU: provides a broad range of services and resources that may differ based on institution, hospital size, and referral base. Geographic setting will impact populations/ disease entities served, available providers and support services, relationships with other PICUs at various levels, and transport program capabilities<br>Tertiary PICU: provides advanced care for many medical and surgical illnesses in infants and children. Provide advanced ventilatory support, access to subspecialities, advanced technologies and services.<br>Quaternary Specialized PICU: provides regional care and serves large populations with a large catchment area. Provides comprehensive care to all complex patients, diagnosis-specific care, specialized equipment and subspecialty care teams. This highest level of PICU has readily available resources as a Children's Surgical Center or Trauma Center.
(Hachey et al., 2020)	Strengthening the approach to oral health policy and practice in Canada	CAN	Dentistry	Application of the Health Impact Pyramid paradigm to guide oral health care in Canada.	Oral health care guidelines include utilizing oral health care providers full scope of practice to reduce inequities and integrate oral health into primary care (screening and referral).	Not Available

Supplementary Table IV: Results of academic and grey literature findings on pediatric standards of care *(continued)*

Author	Title	Ctry	Clinical Service Area	Study Aim/ Purpose	Description of Standard or Guideline	Description of Levels or Tiers of Service
(Kilpatrick et al., 2019)	Obstetric care consensus #9: Levels of maternal care	USA	Women and Newborn Health	To reaffirm uniform definitions, standardized descriptions of maternity facility capabilities and personnel and framework for addressing levels of maternal care. Reaffirm goals of levels of maternal care and clarify definitions, revise criteria.	Levels of maternal care are revised from original 2015 Maternal Care Obstetric Care Consensus to clarify terminology and include more recent data. To standardize a complete and integrated system of perinatal regionalization and risk-appropriate maternal care, this classification system establishes levels of maternal care that pertain to basic care (level 1), speciality care (level 2), subspecialty care (level 3), and regional perinatal health care centers (level 4). Levels of maternal care provide definitions, capabilities, and health care providers for each of the 4 levels of maternal care and for birth centers.	**4 Levels of Care:**<br>Level 1: Basic care for low- to moderate-risk pregnancies with ability to detect, stabilize, and initiate management of unanticipated maternal-fetal or neonatal problems during antepartum, intrapartum, postpartum period until patient can be transferred to higher level of care<br>Level 2: Speciality care for moderate- to high-risk antepartum, intrapartum, or postpartum conditions with specialized care teams<br>Level 3: Subspeciality care for complex maternal medical conditions, obstetric complications, and fetal conditions with subspecialist care teams<br>Level 4: Regional perinatal health care centers with on-site medical and surgical care of the most complex maternal conditions and critically ill pregnant women and fetuses throughout antepartum, intrapartum, and postpartum care
(Kazak et al., 2015)	Psychosocial assessment as a standard of care in pediatric oncology	USA	Pediatric Oncology	Review of standard of care for psychosocial assessment in pediatric cancer.	Psychosocial Standards of Care for Childhood Cancer include routine psychosocial assessments for youth and family members after diagnosis to identify family resources, social support, and premorbid functioning.	Not Available
(Lim et al., 2020)	Standards of care for children in emergency departments: executive summary	CAN	Pediatric Emergency	Summary of standards of care for children in emergency departments (ED)	Standards of care for ED are defined as:<br>1. Integrated services: prehospital, primary care, and hospital services for ED; 2. Child and family-centered care; 3. Initial assessment of ill or injured children: triage models for visual inspection and vital signs; 4. Stabilising and treating ill or injured children; 5. ED staffing; 6. Staff training skills for children of all ages; 7. Equipment supplies and medications for different ages and body weights; 8. Quality and safety: continuous quality improvement; 9. Policies procedures and guidelines defining the goals of the organization and procedures; 10. Information systems and data: information of patient flow, admissions, wait-times. 11. Prehospital care; 12. Mass casualty or patient surges plan for major incidents affecting patient care. 13. Safeguarding children: recognizing and managing children presenting signs of abuse or neglect; 14. Adolescent mental health & substance misuse: provide timely care by experienced staff; 15. Death of a child in emergency: processes after the death of a child 16. Advanced training and academic research.	Not Available

Supplementary Table IV: Results of academic and grey literature findings on pediatric standards of care *(continued)*

Author	Title	Ctry	Clinical Service Area	Study Aim/ Purpose	Description of Standard or Guideline	Description of Levels or Tiers of Service
(National Health Service, 2016)	Paediatric critical care standards for London: level 1 and 2. London: National Health Service	UK	PICU	Review of standard of care for Paediatric Critical Care Level 1 and 2 within a district general hospital environment.	Standards of care are defined through three levels of paediatric critical care. Guidelines include establishing networks of critical care services, governance of minimum quality standards and sharing of best practice, treating children at different developmental stages, and defining paediatric critical care interventions at locations with appropriate resources.	**3 Levels of Paediatric Critical Care**:<br>Level 1: Basic critical care unit<br>Level 2: Intermediate critical care unit in a hospital setting or tertiary setting<br>Level 3: Advanced critical care unit
(Provincial Council for Maternal and Child Health, 2013)	Standardized maternal and newborn levels of care definitions.	CAN	Women and Newborn Health	Review of standard of care for maternal and newborn services.	Guidelines of care include services with care providers competent to provide maternal and newborn care (e.g., resuscitation and stabilization), a clearly established referral path and process to higher/specialized levels of care, established patient transfer protocols, appropriate staff education.	**3 Levels of Maternal and Newborn Care:**<br>Level 1: Low risk maternal and newborn care<br>Level 2: Low-to-moderate maternal and newborn risk<br>Level 3: High risk maternal and newborn care with on-site surgical capability
(Provincial Council for Maternal and Child Health, 2021)	Pediatric levels of care	CAN	General Pediatrics	Update standards of care defined by acuity, medical complexity, and procedural complexity to optimize access to care in geographically vast areas with limited expertise and resources.	Pediatric levels of care definitions are defined by low, medium, high acuity and medical complexity and procedural complexity when determining whether quality pediatric care can be safely provided. Three distinct levels of pediatric care are designated by the degree to which hospitals would be prepared to care for pediatric patients.	**3 Levels of Care:**<br>Level 1: General care for pediatric patients, stabilized before being transferred to a higher level of care<br>Level 2: Emergency care and urgent care and admit patients to a dedicated pediatric unit with a pediatrician. Care for pediatric patients with moderate acuity, complexity, and low procedural complexity<br>Level 3: Care for patients with high acuity, high medical complexity, and high procedural complexity. Emergency departments dedicated to pediatric patients, including intensive care units.
(Rosenberg and Moss, 2004)	Guidelines and levels of care for pediatric intensive care units	USA	PICU	Review of scope of pediatric critical care services and elements of hospital care that are necessary to provide high-quality pediatric critical care.	Pediatric critical care is ideally provided by a PICU that meets Level I multidisciplinary care for a wide range of complex, progressive, and rapidly changing medical, surgical, and traumatic disorders. There are minimum guidelines of care for PICU Level I and Level II including organization and administrative structure, physical facility, personnel, hospital facilities and services, drugs and equipment, prehospital care, quality improvement, training, and continuing education.	**2 Levels of PICU Care:**<br>Level I: Provide care to the most severely ill patient population, multidisciplinary care with specialized care of medical and surgical subspecialists including pediatric intensivists.<br>Level II: Provide stabilization of critically ill children before transfer to another center or avoid long-distance transfers for low complexity disorders. Level II units should be located according to demand or need and in concert with accepted principles of regionalization of medical care. Each Level II unit must have a well-established communications system with a Level I PICU.

Supplementary Table IV: Results of academic and grey literature findings on pediatric standards of care *(continued)*

Author	Title	Ctry	Clinical Service Area	Study Aim/ Purpose	Description of Standard or Guideline	Description of Levels or Tiers of Service
(Rowan-Legg, 2013)	Oral health care for children - a call for action	CAN	Dentistry	Call to action statement advocating for strategies to develop more comprehensive, accessible public dental health policy for Canadian children and youth.	Canadian Pediatric Society Recommendations for Standard of Care include:<br>1. Ensure all children in their respective jurisdictions are afforded equal access to basic treatment and preventive oral care, regardless of where they live or socioeconomic status.<br>2. Ensure every child has a dental home by 1-year of age.<br>3. Support the Canadian Pediatric Society and the Canadian Dental Association recommendations on fluoride supplementation.<br>4. Create leadership positions to represent specific interests of children and youth on oral health issues.<br>5. Develop ongoing surveillance to capture key data and reflect the state of pediatric oral health.<br>6. Prioritize research investigating evidence-based pediatric dental practices and the long-term effects of social determinants on oral health.<br>7. Child health care providers receive appropriate training and continuing education in oral health, with emphasis on early risk assessment and provision of anticipatory guidance.<br>8. A multidisciplinary approach to pediatric oral health care be developed, involving physicians, dentists, hygienists, nurses and schools.	Not Available
(Tasmanian Government, 2016)	Tasmanian role delineation framework and clinical services profile Version 4	AUS	General Pediatrics	Review of clinical services and Tasmanian Role Delineation Framework outlining standard of care for safe and sustainable services.	This framework will enable each clinical specialty to provide a safer patient transition along the full continuum of care and is a planning document to describe the minimum support services, safety standards, skills and competencies, networking arrangements, and other service requirements necessary to provide a service at a specific level to ensure safe and appropriately supported clinical service delivery.<br>Delineating the level of clinical services, not the facilities themselves, consists of core clinical services and clinical support services.	**6 Levels of Service:**<br>Level 1: Low complexity ambulatory care services<br>Level 2: Low complexity inpatient and ambulatory care services<br>Level 3: Low to moderate complexity inpatient and ambulatory care services<br>Level 4: Moderate complexity inpatient and ambulatory care services<br>Level 5: Moderate to high complexity inpatient and ambulatory care services<br>Level 6: High complexity inpatient and ambulatory care services

Supplementary Table IV: Results of academic and grey literature findings on pediatric standards of care *(continued)*

Author	Title	Ctry	Clinical Service Area	Study Aim/ Purpose	Description of Standard or Guideline	Description of Levels or Tiers of Service
(Wiener, Kazak, et al., 2015)	Standards for the psychosocial care of children with cancer and their families: An introduction to the special issue	USA	Pediatric Oncology	Review of 15 standards of care for children with cancer and their families to receive essential psychosocial care.	15 Psychosocial Standards of Care for Pediatric Cancer:<br>1. Assessment of psychosocial healthcare needs<br>2. Monitoring of neurocognitive problems<br>3. Screening in long-term survivorship 4. Access to psychosocial support and interventions<br>5. Assessment of financial need (at diagnosis and ongoing) 6. Parental mental health<br>7. Psychoeducation, information, anticipatory guidance 8. Prep information about invasive medical procedures and psychological intervention 9. Opportunities for social interaction<br>10. Psychosocial support and interventions for siblings 11. Support for school re-entry 12. Adherence to treatment is assessed and monitored 13. Palliative care concepts throughout disease process 14. Psychosocial care after a child's death 15. Open, respectful communication and collaboration among medical and psychosocial providers, patients, and families	Not Available
**Grey Literature**						
(Child Health BC, 2018c)	Children's Medical Services	CAN	General Pediatric	Summary of Tiers of Service Framework and approach for planning and coordinating children's general health services in British Columbia, Canada.	Standards of care are defined within Tiers of Service levels and expected to align with service needs of children living within a geographic area of focus. Tiers of service are differentiated by patient level of acuity and medical complexity (i.e., low, medium, high). Responsibilities and requirements at each tier are divided into clinical services delivered by inpatient, hospital-based, outpatient, and community-based services.	**6 Tiers of Service:**<br>Tier 1: Prevention, primary & emergent health service<br>Tier 2: General health service<br>Tier 3: Child-focused health service<br>Tier 4: Children's comprehensive health service<br>Tier 5: Children's regional enhanced & subspecialty health service<br>Tier 6: Children's provincial subspecialty health service
(Child Health BC, 2018b)	Children's Emergency Medical Services	CAN	Pediatric Emergency	Summary of Tiers of Service Framework and approach for planning and coordinating children's emergency health services in British Columbia, Canada.	Standards of care are defined within Tiers of Service levels and provided to children within emergency department with acute and undifferentiated medical problems or injuries. Emergency department tiers of service was developed by interdisciplinary working group for services provided within a hospital.	**6 Tiers of Service:**<br>Tier 1: Prevention, primary & emergent health service<br>Tier 2: General health service<br>Tier 3: Child-focused health service<br>Tier 4: Children's comprehensive health service<br>Tier 5: Children's regional enhanced & subspecialty health service<br>Tier 6: Children's provincial subspecialty health service

Supplementary Table IV: Results of academic and grey literature findings on pediatric standards of care *(continued)*

Author	Title	Ctry	Clinical Service Area	Study Aim/ Purpose	Description of Standard or Guideline	Description of Levels or Tiers of Service
(Child Health BC, 2018a)	Children's Diabetes Services	CAN	Subspecialty (Diabetes)	Summary of Tiers of Service Framework and approach for planning and coordinating children's diabetes health services in British Columbia, Canada.	Standards of care are defined within Tiers of Service levels and include Tier 1, 4, 5, and 6 for diabetes care. The responsibilities and requirements at each tier are summative. Service levels provided by a given team are expected to align with service needs of children living within the team's geographic area of focus. Diabetes tiers of service provides a common language and methodology to describe responsibilities and requirements at each tier. Responsibilities and requirements at each tier are divided into clinical services delivered by outpatient and inpatient services.	**6 Tiers of Service:**<br>Tier 1: Prevention, primary & emergent health service<br>Tier 2: General health service<br>Tier 3: Child-focused health service<br>Tier 4: Children's comprehensive health service<br>Tier 5: Children's regional enhanced & subspecialty health service<br>Tier 6: Children's provincial subspecialty health service
(Healthy Young Minds in Herts, 2023)	The CAMHS tier system explained	UK	Mental Health	Summary of Tiers of Service framework for planning, commissioning, and delivering mental health services.	Hertfordshire's emotional and wellbeing services for children and young people are commissioned through a tiered system.	**4 Tiers of Service**:<br>Tier 1: Early intervention and prevention provided by schools, children's centres, health visitors, school nurses, GPs, Youth Connexions, helplines and websites for support with external factors affecting wellbeing; with general emotional wellbeing; and for online therapeutic support and self-help.<br>Tier 2: Early help and targeted services through community counselling, counselling or mentoring in schools, education psychologists, education support centres, targeted youth support teams, family support and free online counselling.<br>Tier 3: Specialized mental health services, including eating disorder services. Services include specialist multi-disciplinary approach to children and young people aged 0-19 who have a global learning disability and/or Autistic Spectrum Disorder and their families.<br>Tier 4: Inpatient provision and specialized day and inpatient units, where people with more severe mental health problems can be assessed and treated.

Supplementary Table IV: Results of academic and grey literature findings on pediatric standards of care *(continued)*

Author	Title	Ctry	Clinical Service Area	Study Aim/ Purpose	Description of Standard or Guideline	Description of Levels or Tiers of Service
(International Federation of Emergency Medicine, 2019)	Standards of care for children in emergency departments	INT	Pediatric Emergency	Summary of standards of care for emergency care of children using expert consensus approach.	Recommendations for standards of care are divided by essential and desirable to be a practical tool to help emergency workers and their managers globally, to advocate and motivate for improvements to the emergency care of children in their own facilities. Essential and desirable recommendations are made for integrated service design; child and family centered care; assessment of ill or injured children; stabilizing and treating ill or injured children; staffing of an emergency department; staff training and competencies; emergency department equipment, supplies, medications; quality and safety; policies, procedures and guidelines; information system and data analysis; pre-hospital care; mass casualty incidents and patient surges; safeguarding children and young people; adolescent mental health and substance misuse; death of a child in emergency; advanced training and academic research.	Not Available
(New Zealand Ministry of Health, 2014)	The NZ role delineation model	NZ	General Pediatrics	Clinical services model to differentiate capability of services and complexity between services within, and across District Health Board providers.	The six levels of service complexity extend from community-based services through to the most complex setting. This model requires a service to have an appropriate mix of subspecialties to achieve higher levels of complexity. This model creates specialty service capability levels that can be used to describe and understand pediatric services across the region.	**6 Levels of Service:**<br>**Level 1:** Primary and community-based services provided by primary practitioners. May be in a rural, provincial or urban setting<br>**Level 2:** General and convalescent services, sometimes in rural communities, providing sub-acute care and access to acute services<br>**Level 3:** Specialist services providing acute and elective care to communities<br>**Level 4:** Specialized services with some subspecialisation<br>**Level 5:** Major specialist services with multiple subspecialties and subspecialty support<br>**Level 6:** Supra specialist and definitive care services most complex of any subspecialty

Supplementary Table IV: Results of academic and grey literature findings on pediatric standards of care *(continued)*

Author	Title	Ctry	Clinical Service Area	Study Aim/ Purpose	Description of Standard or Guideline	Description of Levels or Tiers of Service
(New Zealand Ministry of Health, 2012)	Guidance for integrated pediatric palliative care services in New Zealand	NZ	Palliative Care	Provide implementation-focused guidance to improve the integration of palliative care service delivery to children and young people in New Zealand.	Develop guidance on levels of care that are evidence-based, provides analysis of current service delivery, reflects stakeholder involvement, proposes models of service delivery with a focus on implementation, can be implemented at no extra cost to health sector.	**3 Levels of Care:**<br>Level 1: Palliative care approach are appropriately applied by all providers<br>Level 2: General palliative care is provided by professionals with additional training and expertise at an intermediate level.<br>Level 3: Specialist palliative care is provided by providers whose core activity is provision of palliative care services.
(New Zealand Ministry of Health, 2011)	National Plan for Child Cancer Services in New Zealand	NZ	Pediatric Oncology	Role Delineation Model for pediatric oncology and haematology services.	The six levels of role delineation model differentiates between the levels of complexity of services provided by New Zealand's District Health Boards and enables services to be described consistently.	**6 Levels of Service:**<br>Level 1: Primary and emergency care for stabilisation before moving to appropriate higher level of service, no pediatric oncology services<br>Level 2: General pediatric outpatient consultation before moving to appropriate higher level of service<br>Level 3: Specialist pediatrician and health care providers with experience in pediatric oncology, supports outpatient pediatric chemotherapy, disease surveillance and late effects monitoring<br>Level 4: Adult oncology service on site with enhanced laboratory and blood transfusion services and presence of radiation oncology<br>Level 5: Pediatric subspecialty services available on site, supports all forms of cancer treatment, dedicated child/adolescent cancer unit, specialist oncologists and haematologists 24-hour cover<br>Level 6: Pediatric intensive care unit, dedicated child/adolescent palliative care, allogeneic haemopoietic stem cell transplantation
(Queensland Health, 2014)	Medical services—Children's Module Overview	AUS	General Pediatric	Summary of Tiers of Service framework for Queensland general pediatric services.	Guidelines for Tiers of Service framework include description of services provided, service requirements, workforce requirements, and risk considerations. There are required documented processes at each level of medical service with public or licensed private health facility for referral and transfer of patients to/from higher capability level services to provide safe, ongoing care and management of children.	**6 Tiers of Service:**<br>Tier 1: Ambulatory care<br>Tier 2: Ambulatory care and limited inpatient service<br>Tier 3: Inpatient services for single-system disorders and low-acuity medical conditions<br>Tier 4: Inpatient services within a children's ward<br>Tier 5: Stand-alone multidisciplinary teams, specialties, rehabilitation, child protection and developmental services<br>Tier 6: Complex medical services to children on statewide and interstate wide basis, specialist care teams

Supplementary Table IV: Results of academic and grey literature findings on pediatric standards of care *(continued)*

Author	Title	Ctry	Clinical Service Area	Study Aim/ Purpose	Description of Standard or Guideline	Description of Levels or Tiers of Service
(Sutton Council, 2004)	CAMHS Standard, National Service Framework for Children, Young People and Maternity Services	UK	Mental Health	Summary of Tiers of Service framework for planning, commissioning, and delivering mental health services.	Tiers of Service framework ensures commissioning and availability of a comprehensive range of services to meet all the mental health needs of children and young people in an area, with clear referral routes between tiers.	**4 Tiers of Service**:<br>Tier 1: Services provided by practitioners in universal services; including GPs, school nurses, teachers, social workers, youth justice workers and voluntary agencies.<br>Tier 2: Specialists working in community and primary care settings including primary mental health workers, psychologists and counsellors working in GP practices, paediatric clinics, schools, and youth services.<br>Tier 3: Multi-disciplinary teams or services working in a community mental health clinic or child psychiatry outpatient service, providing a specialised service for children and young people with more severe and complex disorders.<br>Tier 4: Essential tertiary level services for children and young people with the most serious problems, such as day units, highly specialised outpatient teams and in-patient units.
(Victoria State Government, 2019)	Maternal and child health program standards	AUS	Women and Newborn Health	Summary of a framework for women and newborn health standards of care.	The framework for the provision of maternal and child health services is to promote health outcomes by providing a comprehensive and focused approach to managing the physical, emotional, or social factors affecting families in contemporary communities. Standards of care support the provision of clinical services and provide a systematic approach to improving service delivery and safety. Six Standards of Care include: 1. Universal Access: responsive and culturally competent service delivery, 2. Optimal Health and Development: health and wellbeing of the child and family, 3. Partnerships and Collaboration: working with mothers and families, other services and organizations within the community, 4. Competent and Professional Workforce: human resource management planning, performance development and continuing education, 5. Responsive and Accountable Service Delivery: leadership and direction, accountability, information systems, 6. Quality and Safety: improving performance, safe practice and environment.	Not Available
